# Exploring Aquaporins in Human Studies: Mechanisms and Therapeutic Potential in Critical Illness

**DOI:** 10.3390/life14121688

**Published:** 2024-12-20

**Authors:** Charikleia S. Vrettou, Vasileios Issaris, Stelios Kokkoris, Georgios Poupouzas, Chrysi Keskinidou, Nikolaos S. Lotsios, Anastasia Kotanidou, Stylianos E. Orfanos, Ioanna Dimopoulou, Alice G. Vassiliou

**Affiliations:** First Department of Critical Care Medicine, School of Medicine, National and Kapodistrian University of Athens, Evangelismos Hospital, 106 76 Athens, Greece; vrettou@hotmail.com (C.S.V.); vasilisiss@gmail.com (V.I.); skokkoris2003@yahoo.gr (S.K.); poupouzas.gewr.kw@gmail.com (G.P.); chrysakes29@gmail.com (C.K.); n.lotsios96@gmail.com (N.S.L.); akotanid@med.uoa.gr (A.K.); stylianosorfanosuoa@gmail.com (S.E.O.); idimo@otenet.gr (I.D.)

**Keywords:** AQP, critically ill, sepsis, ARDS, AKI, ABI

## Abstract

Aquaporins (AQPs) are membrane proteins facilitating water and other small solutes to be transported across cell membranes. They are crucial in maintaining cellular homeostasis by regulating water permeability in various tissues. Moreover, they regulate cell migration, signaling pathways, inflammation, tumor growth, and metastasis. In critically ill patients, such as trauma, sepsis, and patients with acute respiratory distress syndrome (ARDS), which are frequently encountered in intensive care units (ICUs), water transport regulation is crucial for maintaining homeostasis, as dysregulation can lead to edema or dehydration, with the latter also implicating hemodynamic compromise. Indeed, AQPs are involved in fluid transport in various organs, including the lungs, kidneys, and brain, where their dysfunction can exacerbate conditions like ARDS, acute kidney injury (AKI), or cerebral edema. In this review, we discuss the implication of AQPs in the clinical entities frequently encountered in ICUs, such as systemic inflammation and sepsis, ARDS, AKI, and brain edema due to different types of primary brain injury from a clinical perspective. Current and possible future therapeutic implications are also considered.

## 1. Introduction

### 1.1. Aquaporin Localization and Role in Physiology and Disease

Aquaporins (AQPs) are specialized water channel proteins crucial for maintaining cellular homeostasis by regulating water and other small solutes’ transport across cell membranes. Their discovery revolutionized our understanding of how water moves through biological systems. In 1992, Peter Agre and his team identified the first aquaporin, which was later named aquaporin-1 (AQP1), while studying Rh blood group antigens. They noticed an unexplained protein in red blood cell membranes and kidney tissues that facilitated rapid water transport. By isolating and cloning this protein, they confirmed its function as a water channel [1,2,3,4]. This groundbreaking work earned Agre the Nobel Prize in Chemistry in 2003. Aquaporins are now recognized to be essential for processes such as kidney filtration, glandular secretion, and maintaining water balance in cells.

The family of aquaporins consists of at least 13 different isoforms in humans (AQP0–AQP12), each with unique tissue distribution, distinct physiological roles, and regulatory mechanisms. Table 1 summarizes the findings thus far on the distinct tissue distributions and physiological roles of human AQPs [5,6,7,8].

The key physiological functions across AQPs include water homeostasis, exocrine secretion, energy metabolism, neuroprotection, osmoregulation, and detoxification. The importance of AQPs in physiology extends to various pathophysiological processes, such as cell migration, signaling pathways, inflammation, tumor growth, metastasis, and neurological disorders, highlighting their multifunctional nature in maintaining cellular integrity and responding to physiological challenges [5,7,9,10].

In critically ill patients, such as those with sepsis, acute respiratory distress syndrome (ARDS), acute kidney injury (AKI), and acute brain injury (ABI), which are frequently encountered in intensive care units (ICUs) [11,12], water transport regulation is crucial for maintaining homeostasis, as dysregulation can lead to edema or dehydration, with the latter also implicating hemodynamic compromise [13,14,15,16]. Indeed, AQPs are involved in fluid transport in various organs, including the lungs, kidneys, and brain, where their dysfunction can exacerbate conditions like ARDS, AKI, or cerebral edema [17,18,19,20,21,22,23,24,25,26,27,28,29,30,31]. Additionally, altered AQP expression and function can affect patient outcomes by contributing to vascular permeability and interstitial edema [32,33,34]. Moreover, AQP implication in cell migration and the inflammatory response during critical illness can influence patient response in sepsis [35,36]. Finally, in trauma cases, AQPs can contribute to the body’s attempt to maintain fluid balance and restore homeostasis [31,37,38,39,40,41,42,43,44,45,46,47]. Understanding the roles of AQPs in these contexts can aid clinicians in managing fluid therapy more effectively and minimizing complications associated with organ dysfunction.

**Table 1:** Aquaporins: Localization and role in physiology and disease.

Aquaporin	Localization	Function	Role in Critical Illness	References
AQP0	Eye lens, skin, male reproductive system	Acts as both a water channel and a structural protein in the lens. Maintains lens transparency and hydration	Impairment may contribute to cataracts and other ocular complications in critically ill patients	[8,48,49]
AQP1	Kidneys, eye, brain, heart, lung, liver, skeletal muscle, blood cells, and various glands	Facilitates water reabsorption, crucial for urine concentration and fluid balance for maintaining plasma volume and cerebrospinal fluid (CSF) production	Altered expression affects fluid retention, edema, and organ function in critical illness	[8]
AQP2	Kidneys (collecting ducts), ear, stomach, intestines, reproductive systems	Regulated by vasopressin; essential in water reabsorption, especially during dehydration	Dysregulation linked to electrolyte imbalances and water retention complications	[8,49,50]
AQP3	Kidney, skin, immune cells, gastrointestinal tract (salivary and pancreatic secretion), lung, spinal cord	Transports water, glycerol, and small solutes, contributing to systemic fluid balance		[8]
AQP4	Brain, spinal cord, lung, kidney, stomach, skeletal muscle	Regulates osmotic balance in glial cells, critical in brain edema	Key role in cerebral edema management in brain injury and neurological illness	[8]
AQP5	Epithelial tissues (salivary glands, airways), lung, immune cells, pancreas, skin	Facilitates fluid secretion, essential for saliva production and lung function	Altered levels associated with respiratory distress and secretion deficits	[8,51,52]
AQP6	Kidney (intercalated cells), ear, female reproductive system	Transports water and anions, aiding acid-base balance	Potentially impacts acid-base imbalances and renal dysfunction in critical illness	[8,53,54]
AQP7	Adipose tissue, kidney, gastrointestinal tract, heart	Transports glycerol and water, facilitating energy balance by releasing glycerol from adipose tissue for gluconeogenesis	Implicated in metabolic dysregulation and fluid imbalances under critical conditions	[8,55]
AQP8	Liver, pancreas, kidney, gastrointestinal tract	Supports cellular osmoregulation, ammonia detoxification, particularly in the liver, and bile secretion	Dysfunction associated with liver and pancreatic issues in critical illness	[8,56]
AQP9	Liver, immune cells, heart, spinal cord, spleen	Transports water, glycerol, urea; role in metabolic processes	Dysregulation affects immunity, metabolic balance, and organ perfusion	[8,57]
AQP10	Intestine, ear, heart	Facilitates water and solute absorption in intestines	Linked to fluid absorption issues and nutrient transport under stress	[8,57,58]
AQP11	Kidney, heart, gastrointestinal tract, reproductive systems	Associated with renal development and function	Deficiency may contribute to renal and cardiovascular complications	[8,57]
AQP12	Pancreas, female reproductive system	Involved in digestive fluid secretion and pancreatic enzyme secretion regulation	Potential role in pancreatic insufficiency and digestive issues in critical care	[8,59]

In the present review, we discuss the implication of AQPs in the clinical entities frequently encountered in ICUs, such as systemic inflammation and sepsis, ARDS, AKI, and brain edema due to different types of primary brain injury. The role of AQPs is viewed from a clinical perspective. Current and possible future therapeutic implications are included in the discussion.

### 1.2. Aquaporin Structure and Function

**Figure 1:**
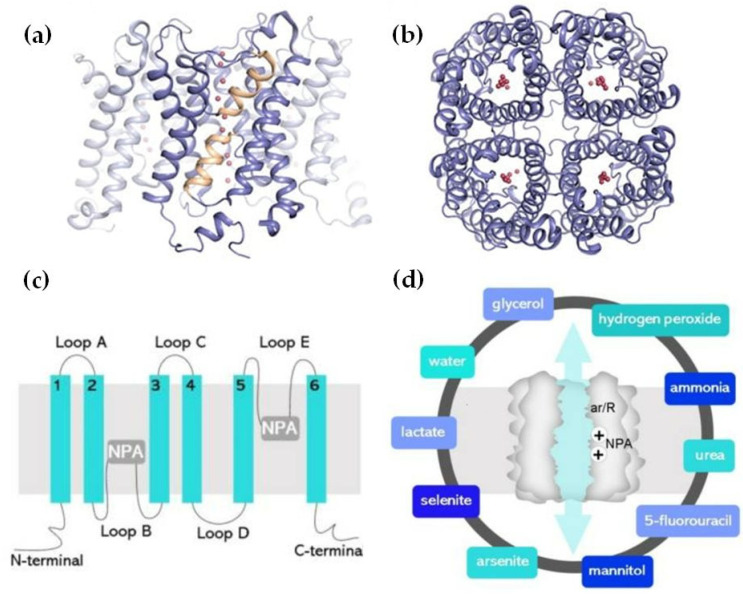
Aquaporin structure. (**a**) AQP side monomer view parallel to the membrane (the two NPA motifs are shown in yellow), (**b**) top view of the AQP tetramer from the extracellular side (water molecules in the pore are shown as red spheres), (**c**) the AQP monomer with the six transmembrane alpha-helices (1–6) connected by five loops extending into the extracellular and cytoplasmic spaces (A–E), and (**d**) illustration of the AQP hourglass-shaped channel, depicting the two selectivity filters (ar/R and NPA), allowing the passage of water molecules and other solutes, as shown, while excluding ions and larger solutes. Abbreviations: AQP, aquaporin; ar/R, aromatic/arginine; NPA, asparagine-proline-alanine. Image adapted from [33,83]. Under Creative Commons CC BY 4.0 license.

The structure of AQPs is characterized by six transmembrane alpha-helices (H1–H6) that form a central pore (Figure 1). These helices are connected by five loops extending into the extracellular and cytoplasmic spaces. This arrangement creates an hourglass-shaped channel, lined with hydrophilic amino acids, allowing the passage of water molecules through the AQP monomer while excluding ions and larger solutes [60,61].

The selectivity of AQPs for water is primarily due to the size and charge of the channel, which is finely tuned to permit the rapid transit of water molecules in response to osmotic gradients [60]. The water transport mechanism through AQPs is facilitated by a unique property known as “single-file diffusion”. Water molecules enter the AQP channel, and are aligned in a single file, enabling rapid movement through the narrow pore [62]. The channel’s structure also contains specific residues playing critical roles in maintaining selectivity and function. For instance, the presence of an asparagine-proline-alanine (NPA) motif, found in the second and fifth loops, is crucial for forming hydrogen bonds with water molecules, thus ensuring the proper conduction of water. Proton exclusion is a critical feature of AQPs. The water molecules inside the channel are oriented by the NPA motifs, disrupting the hydrogen-bonded network required for proton hopping via the Grotthuss mechanism. This ensures that water transport through AQPs does not short-circuit the proton gradient across membranes. Another structural element of the AQP channel important for water selectivity is the “aromatic/arginine (ar/R) selectivity filter”, in which four amino acids, histidine 180, cysteine 189, phenylalanine 56, and arginine 195 (the numbers of these amino acids may differ in different isoforms) come together in the pore and form the part of the pore with the smallest diameter. This narrow pore diameter of AQPs and the presence of positively charged residues within the pore create a size and charge barrier that prevents the passage of charged ions such as sodium, potassium, and hydrogen [60]. (Figure 1). AQPs are specifically designed to exclude ions to maintain osmotic gradients and prevent electrical disturbances across the membrane.

Although each AQP monomer can independently transport water, the channel’s quaternary structure forms a tetramer [63,64,65]. The tetrameric arrangement also forms a central, primarily hydrophobic channel, whose specific function remains unclear (Figure 1). Some believe that this does not act as a pore, whereas others have data showing that it acts as an ion channel that is gated with the loops of the AQP protein being involved in determining when the channel is open or closed [60,66].

AQPs primarily facilitate water transport, but many of them also transport other small solutes like glycerol, nitrate, urea, ammonia, hydrogen peroxide (H₂O₂), carbon dioxide (CO₂), oxygen (O₂), arsenic, antimony, and more, with roles in both physiological functions and in mediating toxicity [6,67,68,69,70,71,72,73,74,75,76]. Human AQPs are, therefore, further classified into orthodox AQPs, aquaglyceroporins, and super aquaporins, with each group facilitating the transport of distinct types of molecules [5]. AQPs 1, 3, 6, 7, 8, 9, and 10 facilitate ammonia transport [77,78]. Aquaglyceroporins, including AQP 3, 6, 7, 9, and 10 also transport urea, potentially linking it to energy metabolism [78]. AQPs 3, 7, 9, and 10 can transport antimonite [70,79,80]. AQPs 0, 1, 4, 5, and 6 are implicated in CO₂ transport, with recent evidence showing that AQP1 also facilitates the transport of O₂ and nitric oxide (NO) [81,82].

### 1.3. Aquaporin Regulation

An important aspect of AQP function affecting their role in pathophysiological processes is their regulation. AQPs are regulated at transcriptional, post-transcriptional, translational, and post-translational levels. These regulatory mechanisms ensure that AQPs respond appropriately to maintain water balance in different tissues and under varying physiological conditions.

At the transcriptional level, hormones such as vasopressin (antidiuretic hormone, ADH) can upregulate AQP expression, especially AQP2 in the kidneys [84,85]. Changes in osmotic gradients are another major factor affecting AQP regulation. Hyperosmolar or hypoosmolar conditions can influence the expression of AQPs like AQP1 and AQP4 in the brain and kidneys [5,86]. Cytokines and inflammatory mediators can modulate AQP gene transcription in response to injury or infection [87,88]. Finally, hypoxic conditions can upregulate or downregulate AQPs, such as AQP1 and AQP4, depending on the tissue and context [89,90,91].

Post-transcriptional modifications include alternative splicing and microRNAs (miRNAs). Different splicing patterns can affect the production of functional AQP isoforms [92], whereas miRNAs can bind to AQP mRNA and suppress their translation, impacting protein levels [93].

At the post-translational level, phosphorylation, ubiquitination, and methylation also play a crucial role in regulating AQPs. For example, phosphorylation at serine residues in the AQP2 protein is essential for its trafficking to the plasma membrane. The dephosphorylation of AQP2 by phosphatases can reverse the effects, promoting the removal of AQP2 from the membrane. Phosphorylation not only controls the localization and membrane abundance of AQPs but can also modulate their water permeability directly [94,95,96,97,98,99,100]. Ubiquitination marks AQPs for degradation, affecting their turnover and cellular abundance, while methylation and/or acetylation can alter protein function or interactions, although this is less studied for AQPs [101].

There are also other regulatory mechanisms. AQPs can be stored in intracellular vesicles and trafficked to the plasma membrane in response to ADH stimulation [102] or can be inserted and removed from the plasma membrane through endocytosis and exocytosis, respectively [103]. AQPs often interact with other proteins, such as anchoring proteins or scaffolding molecules, to stabilize their localization and function [83,104]. Changes in osmotic gradients or mechanical forces can directly influence the gating of AQPs, altering their water permeability [5,86]. Finally, pathological conditions can dysregulate AQP expression, as described in detail below.

**Figure 2:**
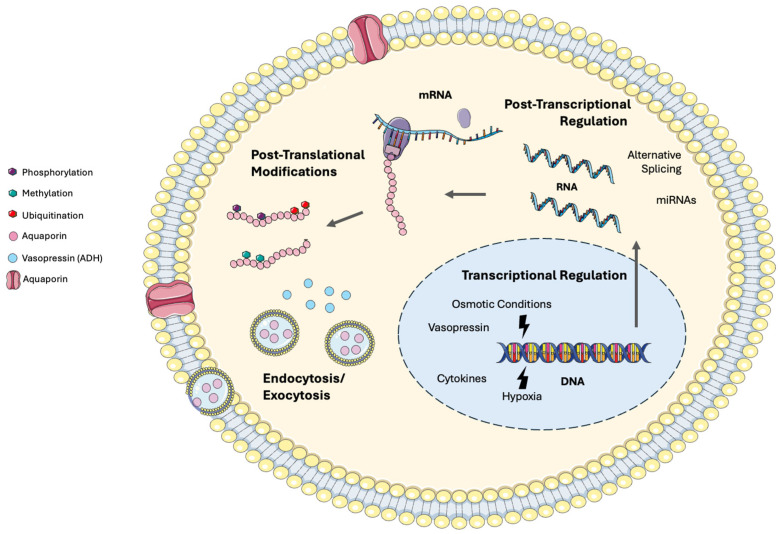
Regulation of aquaporin expression and function. Aquaporin gene expression is tightly regulated by several factors including cytokine levels, hypoxia, cell osmotic conditions, and vasopressin. Following RNA expression, alternative splicing events and regulatory molecules such as micro RNAs (miRNAs), act upon the RNA molecules, thus regulating protein production. A series of post-translational modifications, including phosphorylation, methylation, and ubiquitination determine the function and localization of the formed proteins. AQPs may be alternatively stored in intracellular vesicles to be rapidly transported to the cell membrane upon stimulation. Abbreviations: AQP, aquaporin; miRNAs, micro RNAs.

Figure 2 illustrates the mechanisms that provide a highly coordinated way to control AQP gene expression, protein synthesis, and protein activity. These regulatory mechanisms allow AQPs to respond dynamically to cellular and extracellular environment changes, maintaining homeostasis and adapting to physiological and pathological conditions.

## 2. Aquaporins in Critical Illness

### 2.1. The Role of Aquaporins in ICU Patients with Sepsis

#### 2.1.1. Sepsis

The Third International Consensus (Sepsis-3) currently defines sepsis as “organ dysfunction caused by a dysregulated host response to infection”. In the most severe form of sepsis, septic shock, there is profound circulatory and cellular/metabolic dysfunction. Persistent hypotension despite fluid resuscitation, along with elevated lactate levels, are hallmarks. Multiorgan failure can occur as a result of prolonged hypoperfusion and metabolic disturbances, with death being the likely outcome if untreated. Its pathophysiology includes an exaggerated inflammatory response to an infection leading to endothelium dysfunction, and increased vascular permeability, triggering coagulation abnormalities including microthrombi formation and decreased fibrinolysis [105]. Despite great progress in research and clinical management made in the last three decades, sepsis mortality is still high and, therefore, novel regulatory mechanisms and therapeutic strategies are still needed.

#### 2.1.2. Aquaporin Expression in Human Immune Cells

**Figure 3:**
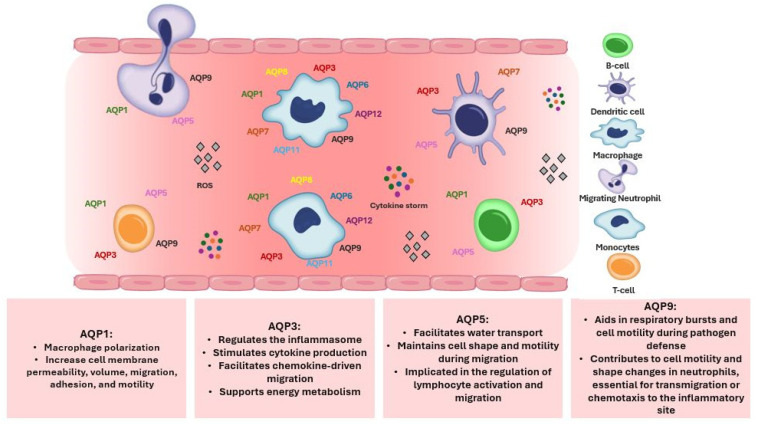
Aquaporin expression in human immune cells and their proposed roles in sepsis. AQP1 is expressed in neutrophils, monocytes, and lymphocytes, contributing to water transport and cell volume regulation during migration and differentiation. AQP3 is expressed in macrophages, lymphocytes, and dendritic cells, facilitating chemokine-driven migration and supporting energy metabolism through glycerol transport. AQP5 is expressed in some immune cells, particularly lymphocytes, neutrophils, and dendritic cells. AQP5 plays a role in facilitating water transport, which is critical for maintaining cell shape and motility during migration. AQP9 is most prominently expressed in leukocytes, aiding in respiratory bursts and cell motility during pathogen defense. It is expressed in neutrophils, macrophages, monocytes, lymphocytes, and dendritic cells. Other AQPs detected at low concentrations in immune cells are AQP6, AQP7, AQP8, AQP11, and AQP12, expressed in monocytes, macrophages, and dendritic cells. AQPs are important regulators of fluid balance/edema, inflammation/immune response, and vascular permeability. During inflammatory conditions, such as sepsis, AQP expression is altered and correlates with disease severity and outcomes. Abbreviations: AQP, aquaporin; ROS, reactive oxygen species. Image adapted from [117].

Aquaporins (AQPs) are expressed in various human immune cells, including lymphocytes, macrophages, neutrophils, and dendritic cells, where they play crucial roles in immune functions such as migration, proliferation, and response to inflammation (Figure 3). AQP1 is expressed in neutrophils, monocytes, and lymphocytes contributing to water transport and cell volume regulation during migration and differentiation [87,106,107,108]. AQP3 is expressed in leukocytes, including lymphocytes, monocytes, macrophages, and dendritic cells, facilitating chemokine-driven migration and supporting energy metabolism through glycerol transport [87,106,107,108,109,110,111]. AQP5 is expressed in some immune cells, particularly lymphocytes, neutrophils, and dendritic cells [106,112]. AQP5 plays a role in facilitating water transport, which is critical for maintaining cell shape and motility during migration. In the immune context, AQP5 has been implicated in the regulation of lymphocyte activation and migration, although its precise mechanisms in immune responses remain less well-characterized compared to AQP3 or AQP9 [106,112]. AQP9 is found most prominently in leukocytes, and aids in respiratory bursts and cell motility during pathogen defense [113]. Immune cells in which AQP9 seems to have a role are neutrophils, macrophages, monocytes, lymphocytes, and dendritic cells [33,87,108,109,114,115,116]. Other AQPs detected at low concentrations in immune cells are AQP6, AQP7, AQP8, AQP11, and AQP12, expressed in monocytes, macrophages, and dendritic cells [87,108,109].

#### 2.1.3. Aquaporins and Sepsis

Aquaporins are emerging as a novel area of research in sepsis, given their roles in regulating fluid balance/edema, inflammation/immune response, and vascular permeability. Their effects seem to be cell and/or tissue and aquaporin-specific (Figure 3). A significant number of experimental studies are available unraveling the underlying pathophysiological mechanisms attributed to aquaporins during sepsis. Clinical studies have shown that the expression of AQPs is often altered during sepsis. In particular, studies have highlighted changes in AQP levels in the lungs, kidneys, and other organs during sepsis, correlating with disease severity and outcomes. This makes AQPs potential biomarkers for sepsis severity or targets for intervention aimed at improving organ function during septic states. However, clinical applications are still in the early stages, and further investigations are required to fully understand their impact on sepsis outcomes.

Detailed analysis of clinical studies related to aquaporins and critical sepsis are presented below. Investigations have focused on the expression of AQP1, AQP3, AQP5, and AQP9 in blood cells. A possible explanation is that these aquaporins are related to immune cell regulation. Most studies have shown that the expression is increased, and, of importance, a number of them raise the possibility that AQP expression is linked to high mortality.

AQP9 is expressed in immune system cells and most prominently in leukocytes. Ishibashi et al. identified the AQP9 cDNA in human leukocytes by polymerase chain reaction. They showed that AQP9 is expressed at the highest levels in polymorphonuclear cells (PMNs), followed by liver, lung, and spleen, suggesting that AQP9 may play a role in the immunological function of leukocytes [114]. The ability of neutrophils to sense and move to sites of infection is essential for defense against pathogens. Matsushima et al. showed that the expression of AQP9 in PMNs is increased in patients with systemic inflammatory response syndrome (SIRS) compared to healthy volunteers. Moreover, they showed that PMNs exhibited morphological and functional changes [115]. Of note, AQP9 is permeable to water and other small molecules such as glycerol, urea, and certain metabolic by-products like lactate. Loitto et al. reported that AQP9 plays an essential role in water influx during the activation of PMNs. They proved that the water influx through AQP9 contributes to cell motility and shape changes in PMNs, which are essential for transmigration or chemotaxis of PMNs to the inflammatory site [118]. Taken together, these suggest that AQP9 may play a significant role in morphological and functional changes in PMNs during SIRS. In contrast, AQP9 expression was not altered during sepsis, and low levels were associated with increased survival [119].

Additionally, Thon et al. also measured AQP3 and showed that AQP3 mRNA expression in whole blood samples from septic patients increased over the course of sepsis, correlated with the lymphocyte count, and most importantly high AQP3 expression was associated with increased survival. Thus, the roles of AQP3 and AQP9 differ in the pathophysiology of sepsis and may be useful biomarkers for sepsis. Furthermore, modulation of their expression might offer a therapeutic option. This study also found that AQP3 correlated with interleukin (IL)-8 levels, suggesting that AQP3 might affect cytokine signaling pathways [119]. AQP3 is expressed in human leukocytes, monocytes, lymphocytes, and dendritic cells [106,107,108,109]. Moreover, AQP3 appears to be important for T-cell function [110,111]. AQP3 seems to regulate the inflammasome, which in turn stimulates cytokine production [108]. Taken together, these suggest that AQP3 has a key role in immune responses. Finally, in a gene expression analysis of five critically ill patients who developed sepsis and septic shock, AQP3 was found downregulated [107]. The AQP3 polymorphism (rs17553719) exhibited an association with 30-day survival in patients with sepsis. Patients with the homozygote CC genotype had lower 30-day survival rate compared to the CT and the TT genotypes, while patients with the CC genotype had higher AQP3 mRNA at study admission compared to the other genotypes [120].

The leukocyte AQP1 expression levels were measured in critically ill patients on ICU admission upon diagnosis of sepsis, and at the occurrence of septic shock. AQP1 was induced in the leukocytes of patients with ICU-acquired sepsis and exhibited higher expression in septic shock. Furthermore, neutrophil AQP1 expression was induced by LPS in vitro [107]. AQP1 was the first mammalian AQP reported to be observed in erythrocytes and renal tubules and was originally named channel-forming integral membrane protein of 28 kDa (CHIP28) [1]. Subsequent studies have demonstrated that it is physiologically distributed in the choroidal plexus, corneal endothelium, pain-processing C-fibers of the spinal cord, and all vascular endothelial cells except in the central nervous system (CNS) [8]. Moreover, AQP1 plays a role in cell migration and angiogenesis in cancer [121]. In the context of sepsis, AQP1 overexpression in leukocytes may increase their cell membrane permeability, increasing their volume and, hence, migration. In addition, in experimental studies, AQP1 emerges as a critical scaffold for a plasma membrane-associated multiprotein complex important for cytoskeleton build-up, adhesion, and motility [122].

A growing number of studies have aimed to investigate the interplay between long non-coding RNAs (lnRNAs) and micro RNAs (miRNAs) in the regulation of aquaporins, and especially their potential role as therapeutic targets in sepsis [34,93]. Non-coding RNAs are non-protein coding RNAs that regulate gene expression on an epigenetic, transcriptional, and post-transcriptional level. The most studied classes of non-coding RNAs are lncRNAs and miRNAs. lncRNAs lack any protein-coding capacity and consist of more than 200 nucleotides, while miRNAs are short non-coding RNAs of 18–24 nucleotides [123,124]. The recent identification of lnRNAs and miRNAs as regulators of aquaporin expression in several conditions has instigated research into the potential role of these molecules in the setting of sepsis.

In one study, septic patients had lower serum lncRNA H19 and AQP1 mRNA levels and higher miR-874 levels compared to healthy controls, showing a negative relationship in septic patients. Based on their results, lncRNA H19 functioned as an AQP1 competitive endogenous RNA (ceRNA) in regulating miR-874, which directly interacted with AQP1 [125]. Similarly, a subsequent study showed that the serum levels of lnRNA H19 were decreased in septic patients compared to healthy controls [126]. The results of these studies propose that lnRNA H19 could be used as a biomarker of early sepsis diagnosis.

The lncRNA cancer susceptibility candidate 2 (CASC2) has been shown to regulate AQP1 expression via the miR-144-3p/AQP1 axis [127]. In a study including 184 septic patients, circulating lncRNA CASC2 levels were found to be decreased compared to healthy controls, while lncRNA CASC2 was associated with disease severity and multi-organ injuries and could be used as a prognostic biomarker [128].

In 2008, upon sequencing of the AQP5 promoter, a novel functional -1364A/C polymorphism was identified and was associated with decreased AQP5 expression. The presence of the C allele was associated with lower mRNA and protein AQP5 expression [129]. Subsequently, the same group studied 154 patients with sepsis and demonstrated that the -1364A/C polymorphism was associated with higher 30-day survival. Thus, they concluded that decreased AQP5 gene expression seemed to enhance survival by reducing the inflammatory burden during sepsis [130].

Epigenetic regulation via DNA methylation also contributes to a differential AQP5 expression in sepsis. More specifically, DNA methylation of the AQP5 promoter results in decreased reporter gene transcription, while demethylation induces AQP5 expression [131]. Rump et al. performed DNA methylation analysis within the AQP5 promoter in 135 septic patients on blood samples collected within the first 24-h after sepsis diagnosis. The methylation analysis revealed that CpG methylation at the AQP5 promoter position “nt-937” was independently associated with an increased risk of death within 30 days. Moreover, non-survivor septic patients had higher AQP5 mRNA expression levels in the blood cells compared to survivors, and, of importance, increased expression was linked to increased risk of death. They concluded that DNA methylation within the AQP5 promoter could act as a possible mechanism for differential AQP5 expression [101].

In a later study, the same group associated the presence of the C-allele of the AQP5-1364A/C polymorphism with a lower gene expression in septic patients, accompanied by a higher methylation level of the AQP5 promoter. Moreover, there was a differential AQP5 promoter methylation in different types of immune cells of septic patients, suggesting that AQP5 promoter methylation could drive genotype-dependent expression [132].

Zheng et al. aimed to investigate the relationship between miR-34b-5p, which has been associated with sepsis-induced organ injury, and AQP2. In the sera of septic patients with AKI miR-34b-5p expression levels were found elevated compared to healthy controls. This study reported miR-34b-5p as a novel biomarker in septic AKI patients, which could regulate sepsis-induced renal injury by inhibiting AQP2 [133].

#### 2.1.4. Proposed Mechanisms of Aquaporin Involvement in Sepsis

Experimental studies have aided in unraveling the underlying pathophysiological mechanisms attributed to aquaporins during sepsis.

(i) Increased Vascular Permeability and Edema

During sepsis, inflammation can disrupt the endothelial barrier, causing fluids to leak from blood vessels into tissues, resulting in edema. AQPs, particularly AQP1 (found in vascular endothelium) and AQP4 (found in the brain and lung), may contribute to this permeability by facilitating water movement, which exacerbates tissue edema in organs like the lungs (ARDS) and brain (cerebral edema) [134,135,136,137,138].

Moreover, studies have shown that AQPs can be upregulated in response to inflammatory mediators such as cytokines, which are elevated during sepsis [87,88,139]. This increase can lead to more water transport across membranes, contributing to the excessive tissue swelling seen in septic patients.

(ii) Contribution to Organ Dysfunction

Aquaporins in the kidneys (notably AQP2 in the collecting duct) are critical for regulating water reabsorption [140]. Sepsis-induced dysregulation of AQP2 can impair water reabsorption, potentially leading to fluid imbalances and worsening renal function [133,141,142,143,144,145].

Aquaporins are also highly expressed in the brain and lung and have been implicated in cerebral and pulmonary edema during sepsis [34,146]. This renders them potential therapeutic targets to reduce brain swelling and improve neurological outcomes in severe sepsis cases. The involvement of aquaporins in brain and lung injury in critically ill patients is discussed further below.

(iii) Inflammatory Modulation

Some aquaporins, like AQP1, may play roles in modulating cytokine production and other inflammatory processes. AQP-mediated changes in cell volume and migration can impact immune cell functions, influencing the systemic inflammatory response characteristic of sepsis [107,121,122].

(iv) Barrier Integrity

In sepsis, aquaporins can influence the tight junctions of cells, which help maintain barrier integrity [147]. Dysregulation in aquaporin expression can thus contribute to the breakdown of barriers in various organs, including the intestines, lungs, and kidneys, leading to further complications in septic patients.

#### 2.1.5. Aquaporins as Therapeutic Targets in Sepsis

Targeting specific AQPs has been proposed as a potential therapeutic strategy in sepsis management. AQP9 has emerged as an attractive therapeutic target. In a murine LPS-induced endotoxic shock model, the AQP9-knockout (KO) mice exhibited longer survival times and decreased inflammation compared to wild-type (WT) mice [148], highlighting the potential of AQP9 as a promising target for the development of new therapies against sepsis.

The sulfonamides methazolamide and furosemide reduced AQP5 expression in REH cells, while methazolamide could also reduce cell migration, suggesting that methazolamide has the potential to be used in sepsis prophylaxis [149].

Experimental AQP3 inhibitors, such as gold-based compounds (e.g., Auphen), have shown promise in preclinical models but are not yet approved for general use [150]. In monocytic cell lines, AQP3 silencing or selective inhibition via Auphen, could revert LPS-priming and decrease the production of pro-inflammatory cytokines, pointing towards AQP3 as a potential target in the inflammatory response process [87].

Overexpression of the long non-coding RNA (lnRNA) FGD5-AS1 in LPS-induced inflammatory animal and cellular models resulted in increased AQP1 levels and reduction of pro-inflammatory cytokines, thus inhibiting the overall inflammatory response [151].

Targeting specific AQPs has also been proposed in sepsis management to reduce edema and improve organ function. For instance, AQP4 inhibitors have been proposed as potential therapeutic targets to alleviate cerebral edema [152,153].

Several studies have explored the effects of various AQP small-molecule inhibitors and how they may attenuate the inflammatory response. However, the clinical application of AQP modulation in sepsis requires further research.

#### 2.1.6. Summary of Aquaporins in Sepsis

Aquaporins play an important role in the pathophysiology of sepsis by regulating water movement and potentially influencing inflammation. They are implicated in the fluid imbalances and edema formation that are prominent in sepsis-related organ dysfunction, particularly in the brain, lungs, and kidneys. Understanding and potentially modulating AQP activity could offer new avenues for therapeutic intervention in sepsis management, although more research is needed to clarify their exact roles and therapeutic viability.

### 2.2. The Role of Aquaporins in ICU Patients with Clinical Acute Lung Inflammation—Acute Respiratory Distress Syndrome (ARDS)

#### 2.2.1. Clinical Acute Lung Inflammation—Acute Respiratory Distress Syndrome (ARDS)

Based on the revised Berlin Definition, acute respiratory distress syndrome (ARDS) is a heterogeneous syndrome characterized by acute onset, distinctive radiographic findings, and increased mortality. It constitutes a severe lung condition characterized by widespread inflammation, increased vascular permeability, and accumulation of fluid in the alveoli, which leads to impaired gas exchange and respiratory failure [154]. Several direct and indirect lung injury etiologies increase the risk of ARDS development. Sepsis, ischemic periods followed by reperfusion, aspiration, toxic inhalation, and radiation are the principal inciting events leading to ARDS [155].

The lung facilitates oxygen and carbon dioxide exchange between air and blood. A liquid layer directly exposed to the gaseous compartment covers the airways and the respiratory zone forming the air–liquid interface. The pulmonary epithelium separates this lining fluid from the extracellular compartment. Maintaining the fluid volume is essential for gas exchange, achieved by balancing fluid transport across the epithelium. While osmosis mainly drives airway fluid transport, filtration may contribute to alveolar fluid accumulation under pathological conditions.

**Figure 4:**
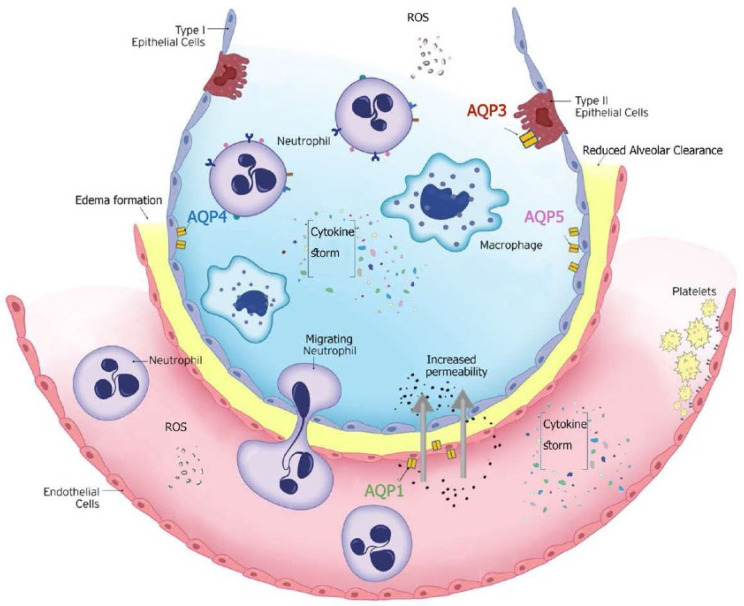
Aquaporins in the human lung. The lung facilitates oxygen and carbon dioxide exchange between air and blood. Maintaining the fluid volume is essential for gas exchange, achieved by balancing fluid transport across the epithelium. Aquaporins (AQPs) are integral membrane proteins that act as water channels, facilitating water transport across cell membranes in response to osmotic gradients. Under physiological conditions, aquaporins usually facilitate fluid management by transporting it out of the alveolar space into the interstitium or capillaries. Τhe main aquaporins expressed in the lung are AQP1, AQP3, AQP4, and AQP5. AQP1 is present in the capillary endothelial cells and controls water movement across the endothelial barrier. AQP3 and AQP4 are found in the airway epithelium and alveolar epithelial cells. AQP5 is expressed in the alveolar epithelial cells type I and is primarily responsible for water transport across the alveolar barrier. Acute respiratory distress syndrome (ARDS) constitutes a severe lung condition characterized by the widespread release of pro- and anti-inflammatory cytokines, oxidative stress (production of reactive oxygen species-ROS), increased vascular permeability, and accumulation of fluid in the alveoli, which leads to impaired gas exchange and respiratory failure. In ARDS, increased water transport by AQPs could contribute to fluid accumulation in the alveoli if the inflammation and barrier dysfunction are uncontrolled, exacerbating pulmonary edema. Disruption of the expression or function of these AQPs, as is often observed in ARDS, compromises the lung’s ability to clear fluid, leading to worsened pulmonary edema. Abbreviations: AQP, aquaporin; ROS, reactive oxygen species. Adapted from [157].

In critically ill patients, particularly those with ARDS, the balance between fluid entry and clearance is disrupted due to increased vascular permeability, impaired alveolar clearance, oxidative stress, and the inflammatory environment of ARDS [156,157,158] (Figure 4). More specifically, inflammatory mediators, such as cytokines [tumor necrosis factor (TNF)-α, IL-1β], increase capillary permeability, leading to fluid leakage into the alveoli. When the process of removing fluid from the alveolar space is disrupted (e.g., through downregulation in response to inflammatory signals), pulmonary edema worsens.

AQPs are integral membrane proteins that act as water channels, facilitating water transport across cell membranes in response to osmotic gradients. This function is crucial for maintaining fluid balance between the alveolar and interstitial spaces and the capillaries. Disruption in alveolar fluid clearance, due to altered functional expression of respiratory AQPs, underscores their pathophysiological importance in respiratory disease associated with pulmonary edema [136,159]. In ARDS and lung injury in critically ill patients, AQPs are crucial for regulating lung water balance, directly impacting the development and resolution of pulmonary edema. Excessive pulmonary edema formation impairs gas exchange, worsens respiratory failure, and has been associated with adverse outcomes in ARDS patients [160].

#### 2.2.2. Overview of Aquaporins in the Lung

AQP1, AQP3, AQP4, and AQP5 are the main aquaporins expressed in the lung. AQP1 is present in the capillary endothelial cells and plays a role in the control of water movement across the endothelial barrier. AQP3 and AQP4 are found in the airway epithelium and alveolar epithelial cells. AQP5 is expressed in the alveolar epithelial cells type I and is primarily responsible for water transport across the alveolar barrier (Figure 4). The specific localization of AQPs to both endothelial and epithelial cells in alveolar tissue suggests a role for these proteins in fluid movement between the airspace, interstitial, and capillary compartments. Indeed, data from AQP knock-out mice provide evidence for the specific involvement of AQP1 in capillary–airspace fluid movement [134,161]. The first data on the involvement of the AQPs in lung water transport came from experimental models using AQP1 and AQP4 knock-out mice [134,161]. AQP1 null mice had reduced water permeability between airspace and capillary compartments. The results obtained from these models showed that osmotically driven water transport across lung microvessels occurs transcellularly via AQP1 water channels and that the microvascular endothelium is a significant barrier for airspace–capillary osmotic water transport. The authors concluded that AQP1 facilitates hydrostatically driven lung edema but is not required for active near-isosmolar absorption of alveolar fluid. A year later, using AQP5 knock-out mice, Ma and coworkers demonstrated that AQP5 is responsible for most of the water transport occurring across the apical membrane of type I alveolar epithelial cells. Alveolar fluid clearance was unhindered in AQP5-null mice indicating that high alveolar water permeability is not required for active, near-isosmolar fluid transport [162]. The same group used mice deficient in each of the three principal lung aquaporins, AQP1, AQP4, and AQP5, to test the hypothesis that aquaporins are important in neonatal lung fluid balance, adult lung fluid clearance, and formation of lung edema following acute lung injury [163]. They found that despite aquaporins’ role in osmotically-driven lung water transport, they are not required for the physiological clearance of lung water in the neonatal or adult lung, or the accumulation of extravascular lung water in the injured lung [163]. These findings support the expected function of AQPs in active fluid absorption and secretion, but they suggest that the requirement for AQP-facilitated water transport depends on the rate of water transport [136,164].

#### 2.2.3. Aquaporins and ARDS

The functional characterization of AQPs, in addition to their tissue- and cell-specific distribution has garnered considerable scientific interest, particularly in understanding their role in pathological conditions, including ARDS and lung injury in critically ill patients [136]. Dysregulation of AQPs has been linked to the pathophysiology of ARDS. During lung injury and inflammation, changes in aquaporin expression and function can either worsen or improve the severity of fluid accumulation. Moreover, oxidative stress and the inflammatory environment of ARDS can directly impact aquaporin function [88,165].

To the best of our knowledge, the first study to report enhanced AQP1 expression levels in ARDS patients was performed by Lai et al. [166]. The study focused on post-mortem lung tissue samples from ARDS patients. Tissue samples were collected within 48-h from the time of death and immediately after the autopsy. Prior to ARDS development, patients were healthy, while the causes of ARDS included trauma, sepsis, stroke complicated by multi-organ failure, and burns. AQP1 was found to be constitutively expressed in the alveolar endothelium. Compared to lung tissue samples from age- and sex-matched non-smoking patients who had died of non-pulmonary diseases in the ICU, AQP1 expression was found to be mildly elevated (33%) in the alveolar capillary endothelium of ARDS patients. Based on these findings, they suggested that AQP1 upregulation could be, to a minor extent, a potential contributing factor of alveolar flooding in ARDS.

In a genomic expression analysis of five polytrauma, initially non-septic patients who were admitted to the ICU, two of whom developed sepsis and three septic shock and ARDS, many genes were found to be upregulated. To validate these genes, Vassiliou et al. corroborated their results with published expression profiling studies using animal models of LPS-induced and aseptic lung injury. This analysis revealed three genes that were commonly dysregulated in both human patients with severe sepsis and ARDS and in experimental models, among them AQP1 [107].

A study performed on mechanically ventilated critically ill COVID-19 patients with respiratory failure due to viral pneumonia showed that serum AQP1 levels were higher in the patient group compared to the control group of 45 healthy individuals with negative PCR tests for SARS-CoV-2 and no chronic disease [167].

The AQP5 promoter -1364A/C polymorphism has also been studied in the context of ARDS. DNA was extracted from whole blood of 136 ARDS patients, and samples were genotyped for the AQP5-1364A/C SNP. The results indicated that the patients carrying the AQP5 AA genotype showed decreased recovery from AKI and increased mortality. Patients carrying the AQP5 AC genotype presented with significantly higher AKI recovery rates on day 30 compared to AA carriers. Hence, decreased AQP5 expression seems to be associated with increased recovery from AKI in ARDS patients [168]. Using the same pool of 136 patients with ARDS induced by either bacterial pneumonia or primary extrapulmonary sepsis with a secondary bacterial pneumonia, Rahmel et al. also investigated whether the AQP5-1364A/C SNP is associated with pulmonary inflammation and survival in ARDS. Based on their results, the presence of the AA genotype of the AQP5-1364A/C polymorphism was associated with aggravated pulmonary inflammation, as indicated by the elevated bronchoalveolar lavage protein and leukocyte concentrations, while ARDS patients with the C-allele of the AQP5-1364A/C promoter polymorphism had attenuated pulmonary inflammation and higher 30-day survival. However, the study was unable to define the mechanisms by which the AA and AC/CC genotypes contribute to mortality in ARDS since it omitted quantitative histochemistry and pulmonary biopsies [27].

Xie et al. analyzed the differentially expressed genes in the mRNA expression profile dataset GSE32707, which included mRNA expression data from whole-blood samples of 33 ARDS patients and 34 healthy control individuals. While the initial results of the analysis showed increased AQP9 mRNA expression in ARDS patients, a subsequent validation analysis in a different group of ARDS patients and healthy individuals did not confirm these findings [169].

The involvement of lncRNA-5657 in sepsis-induced ARDS has also been investigated. lncRNA-5657 expression was measured in the bronchoalveolar lavage fluid (BALF) cells of 15 patients with sepsis-induced ARDS. Compared to non-septic and non-ARDS patients, lncRNA-5657 BALF expression was found significantly elevated in sepsis-induced ARDS patients. Moreover, sepsis-induced ARDS patients also had higher levels of TNF-α and IL-1β. Based on the above findings and in vitro and in vivo data, they suggested that lncRNA-5657 was involved in the development of sepsis-induced ARDS [170]. While lncRNA-5657 has not been demonstrated to regulate aquaporin expression in experimental sepsis-induced ARDS, a connection between lncRNA-5657 and AQP4 has been identified in sepsis-associated encephalopathy (SAE) [171,172].

Data from an in vivo LPS-induced ALI model supported that miR-126-5p had a protective role, maintaining the expression of AQP1 [173]. Recently, Mao et al. investigated the miR-126-5p expression within 24 h of ICU admission in the plasma of 120 septic patients, 60 of whom had ARDS. Based on their results, miR-126-5p plasma levels were reduced in the septic patients with ARDS compared to the septic patients without ARDS and the healthy controls, while miR-126-5p could differentiate between sepsis patients with and without ARDS. Moreover, miR-126-5p expression negatively correlated with various systemic inflammatory markers, while positively correlated with immune function indicators, including immunoglobulins and T-cells in patients with sepsis-induced ARDS [174].

#### 2.2.4. Proposed Mechanisms of Aquaporin Involvement in ARDS

Data from experimental studies have provided insight into how AQPs might be involved in ARDS.

(i) Role in Pulmonary Edema Formation

In ARDS, the integrity of the alveolar–capillary barrier is disrupted due to inflammation, leading to increased permeability and leakage of fluid into the alveolar space (pulmonary edema). Aquaporins, particularly AQP1 (in endothelial cells) and AQP5 (in alveolar epithelial cells), are involved in the water movement across the alveolar–capillary barrier. Under physiological conditions, they usually facilitate the management of this fluid by transporting it out of the alveolar space into the interstitium or capillaries. Hence, increased water transport by AQPs could contribute to fluid accumulation in the alveoli if the inflammation and barrier dysfunction are uncontrolled, exacerbating pulmonary edema. On the other hand, when the expression or function of these AQPs is disrupted, as is often observed in ARDS, the lung’s ability to clear fluid is compromised, leading to worsened pulmonary edema. This effect has been observed in lung injury models using lipopolysaccharide (LPS) exposure, which suppresses AQP1 expression and exacerbates the fluid imbalance within the lungs [29,173,175,176,177,178,179,180,181,182]. Interventions that counteract AQP1 downregulation have shown potential in mitigating these effects by improving fluid regulation and reducing inflammatory cytokine levels [183,184].

(ii) Role in Pulmonary Edema Clearance

Conversely, efficient clearance of excess fluid from the alveolar space is critical for the resolution of ARDS. AQPs also play a role in clearing excess fluid from the alveoli. Alveolar epithelial cells use AQP5 to help reabsorb water from the alveolar space, transferring it into the interstitial and vascular compartments. Impairment or downregulation of AQPs, especially AQP5, has been observed in experimental lung injury models, resulting in impaired alveolar fluid clearance and contributing to respiratory failure. AQP5 expression has been shown to decrease in alveolar epithelial cells during experimental inflammatory conditions, reducing the ability to clear alveolar fluid [29,175,176,177,178,179,180,182,185,186,187,188].

Impairment in alveolar fluid clearance due to altered functional expression of respiratory AQPs highlights their pathophysiological significance in pulmonary edema-associated respiratory illness.

(iii) Role in the Inflammatory Response

AQPs have been implicated in modulating inflammation in the lungs. Studies suggest that the expression of certain AQPs is altered in response to pro-inflammatory cytokines (e.g., TNF-α, IL-1β), which are abundant in ARDS [87,88]. Changes in AQP expression may affect not only water transport but also cellular processes like migration of inflammatory cells and maintenance of the alveolar epithelial barrier, influencing the overall course of ARDS [164].

(iv) Oxidative Stress and Injury

Oxidative stress, a hallmark of ARDS, can impact AQP function. Reactive oxygen species (ROS) generated during inflammation and lung injury can modify AQPs, leading to changes in their permeability and function [88]. This may further contribute to edema and impaired fluid clearance.

In summary, aquaporins, especially AQP1 and AQP5, are involved in the pathophysiology of ARDS by regulating fluid transport and maintaining the balance between edema formation and clearance. Their dysregulation due to inflammation or injury can exacerbate alveolar fluid accumulation and contribute to respiratory distress.

#### 2.2.5. Aquaporins as Therapeutic Targets in ARDS

Due to their role in water transport and edema formation, AQPs have been proposed as potential therapeutic targets for managing lung injury in ARDS. The modulation of AQP function by enhancing the activity or expression of AQPs could improve alveolar fluid clearance and reduce pulmonary edema. Repurposed FDA-approved drugs like Niclosamide look promising, as they have been shown to increase AQP5 abundance and reduce its degradation, which could help to counteract water transport impairments that often exacerbate lung injury. Moreover, high-throughput screenings of drug libraries have identified compounds that promote AQP5 stability, suggesting a potential therapeutic avenue for enhancing AQP5 expression in lung injury management [189]. Approaches to increase AQP5 levels or activity have been investigated in animal models but have yet to translate into clinical therapies [187,188].

On the other hand, where overactive AQPs contribute to excess water influx into the alveoli, aquaporin inhibitors could be used [165]. Therefore, AQP inhibitors could be explored to reduce water movement into the lung during the early stages of injury. This approach is still experimental and requires further research to assess its safety and efficacy.

A recent review by Lotsios et al. extensively refers to the experimental therapeutic approaches with prophylactic effects against sepsis-induced lung injury [34].

Finally, gene therapy targeting AQPs to restore their normal function or the development of small molecules that modulate the activity of aquaporins could represent future treatment options for ARDS. These therapies would aim to correct the fluid imbalances, a hallmark of the disease.

AQP-based therapies are still experimental, but modulation of these channels may offer new strategies for treating ARDS and other forms of lung injury in critically ill patients. Understanding the precise role of aquaporins in ARDS remains an active area of research. Their importance in lung water regulation suggests that they could be key to developing novel therapeutic interventions for this devastating condition.

#### 2.2.6. Summary of Aquaporins in ARDS

Aquaporins, particularly AQP1 and AQP5, are critical in regulating water transport in the lungs. Their dysregulation in ARDS leads to impaired fluid clearance, exacerbating pulmonary edema and worsening respiratory failure. AQPs contribute significantly to the development and resolution of pulmonary edema (excess fluid in the lungs), which impairs gas exchange and worsens respiratory failure in critically ill patients. A reduction in the expression or function of specific aquaporins, such as AQP1 or AQP5, can impair fluid clearance from the alveoli, leading to ongoing pulmonary edema. Conversely, overexpression or increased activity of certain aquaporins may cause fluid imbalance by allowing excessive fluid to enter the lung tissue or alveolar space.

Although research on targeting aquaporins as a therapeutic strategy is still in its early stages, they hold promise for future interventions aimed at improving fluid management in ARDS and other critical lung injuries.

### 2.3. The Role of Aquaporins in ICU Patients with Acute Kidney Injury

#### 2.3.1. Acute Kidney Injury (AKI)

Acute kidney injury (AKI) impacts 30–60% of critically ill patients, contributing significantly to acute morbidity and mortality [190]. Emerging evidence highlights that the consequences of AKI extend beyond the immediate phase, increasing risks for chronic kidney disease progression, cardiovascular complications, recurrent AKI episodes, and long-term mortality [191]. The Kidney Disease: Improving Global Outcome (KDIGO) workgroup proposed a consensus definition and staging system for clinical practice (the KDIGO definition) that relies on the increase of serum creatinine (of at least ≥0.3 mg/dL within the first 48 h, or ≥1.5 times the baseline value within 7 days) and/or the presence of oliguria (<0.5 mL/Kg/h for at least 6 h), both surrogate markers of glomerular filtration rate [192].

#### 2.3.2. Localization and Physiology of AQPs in the Kidney

AQP1 is a highly selective water channel found in the apical and basolateral plasma membranes of the proximal tubule, the descending thin limbs of Henle, and the descending vasa recta, where it facilitates water reabsorption [193]. Mice lacking AQP1 exhibit polyuria, underscoring its essential role in creating hypertonic conditions [194,195].

AQP2 is a crucial channel protein for regulating urine concentration, located on the apical membrane of principal cells in the collecting duct [196]. Its function in water reabsorption is largely regulated by arginine vasopressin, which increases intracellular cyclic adenosine monophosphate production and promotes serine phosphorylation to facilitate AQP2 trafficking to the plasma membrane [197,198,199]. Normal expression of AQP2 on the apical plasma membrane is essential for renal urine concentration and overall body water balance. AQP2 gene deletion or mutation leads to severe water imbalance and can result in nephrogenic diabetes insipidus [24].

AQP3 facilitates the transport of glycerol and hydrogen peroxide across the cell membrane, influencing intracellular signaling pathways that impact key cellular functions, including proliferation, apoptosis, and migration [200]. AQP7, expressed in the brush border of the S3 segment of the proximal tubule, plays a significant role in metabolism by regulating glycerol transport. While impaired AQP7 expression minimally affects water permeability in proximal tubules, it is linked to serious metabolic disorders, such as obesity and insulin resistance [201]. AQP8 enables bidirectional water and hydrogen peroxide transport across biological membranes [202]. AQP11 is uniquely located in the endoplasmic reticulum membrane of the proximal tubular cells. Interestingly, AQP11 knockout mice develop uremia due to polycystic renal disease [203].

**Figure 5:**
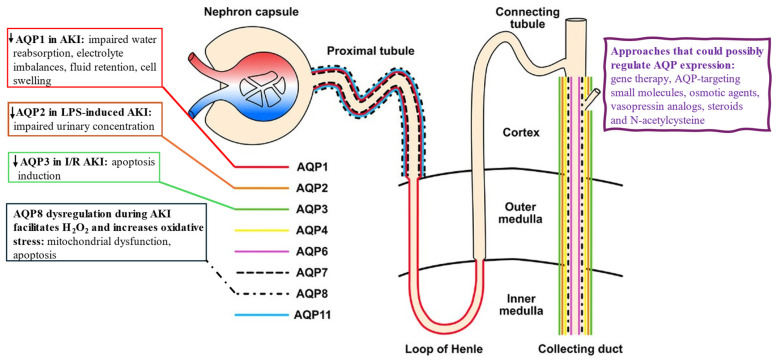
Aquaporins in the human kidney. Aquaporins (AQPs) play a critical role in kidney function by regulating water transport across membranes, crucial for urine concentration and maintaining fluid balance. AQP1 facilitates water reabsorption in the proximal tubules and descending limb of the loop of Henle, while AQP2 is key in the collecting ducts, regulated by antidiuretic hormone (ADH) to concentrate urine. AQPs 3 and 4 support water exit from the collecting duct cells to the bloodstream. AQP7 is expressed in the brush border of the S3 segment of the proximal tubule and plays a significant role in metabolism by regulating glycerol transport. AQP8 enables bidirectional water and hydrogen peroxide transport across biological membranes, while AQP11 is uniquely located in the endoplasmic reticulum membrane of proximal tubular cells. Dysfunction of AQPs can lead to conditions like nephrogenic diabetes insipidus or fluid imbalances. Panels on the left show proposed mechanisms of aquaporin involvement in AKI. The panel on the right shows approaches that could regulate AQP expression. The downward arrows designate decreased expression. Abbreviations: AKI, acute kidney injury; AQP, aquaporin; H_2_O_2_, hydrogen peroxide; I/R, ischemia/reperfusion; LPS, lipopolysaccharide. Image reproduced from [24]. Aspects of this image were partially adapted. Under Creative Commons CC BY 4.0 license.

Figure 5 depicts the localization and function of the aquaporin members in the kidney.

#### 2.3.3. Aquaporins and AKI

Urine AQP2 levels increase in several clinical conditions, such as the syndrome of inappropriate secretion of antidiuretic hormone [204], cirrhosis [205], pregnancy [206], and diabetic nephropathy [207]. On the contrary, histological analysis revealed loss of AQP2 expression in the collecting ducts in patients with elevated bilirubin and cholemic nephropathy (CN), and consequently, the loss of AQP2 in such patients might be the result of toxic effects of cholestasis and in part be responsible for the impairment of renal function [208].

Given the potential diagnostic role for AKI, Chan et al. [209] aimed at determining whether urine AQP2 can predict AKI in patients with acute decompensated heart failure. They conducted a prospective, observational study in a coronary care unit, including 189 patients. AKI was diagnosed in 69 (36.5%) patients, and the median urine AQP2 levels were 61.5 ng/mL and 30.9 ng/mL, respectively, in the AKI and non-AKI groups (*p* < 0.001). Urine AQP2 remained significantly associated with the risk of AKI despite adjustment for other covariates. Furthermore, the area under the receiver operating characteristic curve of urine AQP2 for AKI diagnosis demonstrated an acceptable value (0.795).

In humans, an altered AQP5 expression is linked with a common single nucleotide polymorphism (SNP; -1364A/C; rs3759129) in the AQP5 gene promoter [129]. Substitution of cytosine for adenosine at position -1364 is associated with decreased AQP5 expression [129] and has a significant impact on survival in patients suffering from sepsis [130]. Another study assessed the hypothesis that the AQP5-1364A/C promoter polymorphism is associated with the duration and recovery of AKI in patients with ARDS. One hundred thirty-six patients with ARDS [79 males (58%), 57 females (42%), mean age: 43.7 years)] were prospectively included. ARDS developed in 110 cases (81%) by bacterial pneumonia and 26 cases (19%) by primary extrapulmonary sepsis with secondary bacterial pneumonia leading to ARDS. The ARDS patients were assigned to two groups (AA genotype vs. AC/CC genotype) depending on the -1364A/C polymorphism in the AQP5 gene promoter. The primary end point was AKI presence on day 30 of ICU stay. Homozygous AA genotypes (57%) showed an increased prevalence of AKI compared to AC/CC genotypes (24%), *p* = 0.001. Furthermore, the AA genotype proved to be a strong, independent risk factor for predicting AKI persistence after adjustment for confounders (odds ratio: 3.35; 95–confidence interval: 1.2–9.0; *p* = 0.017) [168].

#### 2.3.4. Proposed Mechanisms of Aquaporin Involvement in AKI

Experimental studies have shed light on how AQPs may play a role in AKI (Figure 5).

(i) Water Reabsorption and Urine Concentration

AQP1, AQP2, AQP3, and AQP4 are predominantly expressed in the kidneys and play crucial roles in water reabsorption, urine concentration, and maintaining water–electrolyte balance, as described earlier. AQP1, which is highly expressed in the proximal tubule and the descending limb of the loop of Henle, supports passive water reabsorption. In cases of AKI, AQP1 dysfunction or downregulation can disrupt this process, leading to impaired water reabsorption, electrolyte imbalances, and fluid retention [193]. Regarding AQP2, studies show that its expression is downregulated in lipopolysaccharide (LPS)-induced AKI, contributing to impaired urinary concentration during sepsis [210]. Notably, downregulation of AQP2 and sepsis-induced AKI progression were alleviated by reduced activation of nuclear factor kappa B (NF-κB) signaling pathways [211].

(ii) Inflammation and Oxidative Stress

In rats with LPS-induced AKI, AQP1 expression is significantly upregulated in the kidneys, making it a valuable biomarker for diagnosing septic AKI [212]. Additionally, higher levels of miR-144-3p have been linked to decreased AQP1 expression, potentially affecting kidney function during LPS-triggered systemic inflammation [213]. Interestingly, AQP1 also has a protective function in LPS-induced AKI by promoting M2 macrophage polarization [214]. Another study suggests that AQP1 helps protect against AKI by modulating the inflammatory response, reducing apoptosis, and attenuating fibrosis through downregulation of P53 in septic AKI or LPS-induced HK-2 cells [215]. Furthermore, AQP8 facilitates hydrogen peroxide (a reactive oxygen species) transport, and its dysregulation can heighten oxidative stress in renal tubular cells, leading to cell damage, mitochondrial dysfunction, and apoptosis during AKI [202].

(iii) Tubular Cell Apoptosis and Necrosis

Lei et al. demonstrated that AQP3 knockdown exacerbates kidney injury by inhibiting mitogen-activated protein kinase (MAPK) signaling and increasing apoptosis in ischemia/reperfusion (I/R) injury in mice [197]. Reduced AQP3 levels may negatively affect renal cell viability after I/R injury, primarily through apoptosis induction. Presumably, increased permeability of AQP-mediated water channels in response to injury can lead to cell swelling or shrinkage, which triggers programmed cell death pathways. In severe AKI cases, excessive water influx and subsequent cell lysis can result in necrosis, intensifying inflammation, and kidney damage [216].

(iv) Renal Ischemia/Reperfusion-Induced AKI

Renal I/R injury is a common cause of AKI, and research has shown a strong association between AQP expression and I/R-induced AKI. For instance, alterations in urine AQP2 levels have been reported in animal models of I/R-induced AKI [217]. Notably, erythropoietin treatment prevented the downregulation of AQPs and sodium transporters in renal I/R injury, and this may play a critical role in improving I/R-induced urinary concentrating defects and impairment of tubular sodium reabsorption [218].

(v) Edema Formation

Histological signs of necrosis are typically more common in proximal tubules than in distal tubules. This discrepancy may be due to the relatively high hydraulic conductivity of proximal tubule membranes, which promotes swelling secondary to sodium influx. AQP1, the primary water channel in the proximal tubule, plays a significant role in water reabsorption and may thus contribute to cell swelling [216].

#### 2.3.5. Aquaporins as Therapeutic Targets in AKI

As discussed above, AQPs are vital water channel proteins involved in kidney function, making them promising therapeutic targets in AKI, by addressing fluid imbalance and cellular damage [145]. Modulation of AQPs, such as restoring AQP1 and AQP3 levels or function or stabilizing AQP2 to enhance water reabsorption, could prove beneficial in the treatment of loss-of-function aquaporin diseases such as nephrogenic diabetes insipidus (NDI), among others [219,220]. Indeed, AQP1, AQP2, AQP3, and AQP4-null mice exhibit diuresis, so restoration of these AQPs shows potential to treat NDI, while their inhibitors are predicted to be novel diuretics [24].

Gene therapy approaches could also regulate AQP expression to support tissue recovery or mitigate inflammation. Pharmacological agents, such as AQP-targeting small molecules, osmotic agents, and vasopressin analogs, offer potential for modulating AQP activity. Anti-inflammatory and antioxidant therapies, like steroids and N-acetylcysteine, could stabilize AQPs and reduce oxidative stress [24].

AQP1 and AQP2 levels in the urine (uAQP1 and uAQP2) have been shown to reflect their respective renal expression levels. Urine levels decrease significantly in animal models of AKI, including gentamicin [221] and I/R-induced AKI rats [217], while studies have reported that AQP5 levels are significantly upregulated in the collecting ducts of patients with diabetic nephropathy (DN) [207,222,223], suggesting that urine levels of these AQPs might be novel noninvasive biomarkers to diagnose DN. It has also been suggested that monitoring AQP levels as biomarkers might guide treatment and track AKI progression [51].

Despite these promising avenues, challenges remain in ensuring specificity, safety, and effective delivery systems, necessitating further research to translate these strategies into clinical practice.

#### 2.3.6. Summary of Aquaporins in AKI

AQPs are expressed in the kidneys and play crucial roles in water reabsorption, urine concentration, and maintaining water–electrolyte balance. Preclinical studies hint at the crucial roles AQPs might play in the development and evolution of AKI. However, there is a lack of clinical studies on their role in the pathogenesis as well as treatment of AKI syndromes in humans, especially in the ICU setting.

### 2.4. The Role of Aquaporins in ICU Patients with Acute Brain Injury

#### 2.4.1. Acute Brain Injury (ABI)

Acute brain injury (ABI), such as traumatic brain injury (TBI), subarachnoid hemorrhage (SAH), acute ischemic stroke (AIS), and intracerebral hemorrhage (ICH), are prevalent in intensive care units (ICUs) due to their severe and life-threatening nature. These conditions often lead to significant neurological deficits and systemic complications, including elevated intracranial pressure, cerebral edema, and impaired cerebral perfusion, necessitating comprehensive monitoring and intervention [224]. Effective management of ABI patients in the ICU requires a multidisciplinary approach that incorporates advanced neuroimaging techniques, hemodynamic support, and strategies aimed at preventing secondary brain injury. Despite progress in critical care practices, patient outcomes remain varied, highlighting the ongoing need for research into the underlying mechanisms and optimal therapeutic approaches for this vulnerable group. Prompt detection and targeted treatment of complications are essential for enhancing survival rates and functional recovery in ABI patients [225].

#### 2.4.2. Localization and Physiology of Aquaporins in the Brain

**Figure 6:**
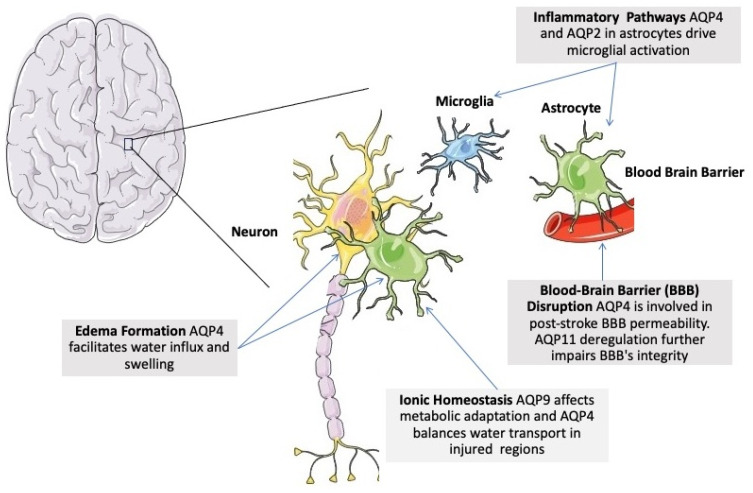
Main pathophysiological roles of aquaporins in acute brain injury. Aquaporins (AQPs) are pivotal in the pathophysiology of acute brain injury, and their role extends across key processes such as cerebral edema formation, blood–brain barrier regulation, and inflammatory response modulation. AQP4 has emerged as a central player, influencing water homeostasis and edema resolution, while other aquaporins, such as AQP1, AQP2, and AQP9, contribute to diverse pathophysiological mechanisms, including immune cell migration and metabolic adaptation. Aspects of this figure were adapted with permission from Servier Medical Art library, available under Creative Commons license.

Under physiological conditions, AQPs are strategically expressed in the brain to maintain water homeostasis and facilitate cerebrospinal fluid (CSF) dynamics [226] (Figure 6). AQP1 is found in the choroid plexus, where it plays a key role in CSF production by enabling water transport across epithelial cells. AQP1-knockout mice exhibit impaired CSF production due to the lack of water transport in the choroid plexus [226]. AQP2 is expressed in astrocytes [227]. AQP4, the most abundant AQP in the brain and is highly expressed in astrocyte end-feet surrounding blood vessels, the glia limitans, and ependymal cells, where it regulates water exchange between the brain parenchyma and blood. AQP4 is also essential for the glymphatic system, which facilitates waste clearance during sleep. AQP4-null mice demonstrate significant alterations in water homeostasis and astrocytic function [9,135,228,229]. In models of cytotoxic edema (e.g., ischemic stroke), these mice show reduced brain swelling, suggesting a protective effect against water influx into the brain parenchyma [228,230,231,232]. Conversely, in cases of vasogenic edema (e.g., trauma or tumors), AQP4-null mice have impaired water clearance, exacerbating fluid accumulation [233,234]. AQP9 is expressed in astrocytes and some neurons, particularly in metabolically active regions, allowing the transport of small solutes like glycerol and lactate to support energy metabolism [235]. Together, these AQPs contribute to the brain’s water balance, metabolic support, and waste clearance under normal physiological conditions [92,230].

#### 2.4.3. Aquaporins and ABI

AQP4, the most abundant aquaporin in the central nervous system, is particularly involved in the regulation of brain edema and the response to traumatic brain injury (TBI), including subdural hematomas (SDH) and acute ischemic stroke (AIS). Genetic variations in AQP4 have been linked to clinical outcomes in TBI. A study by Dardiotis et al. identified several single nucleotide polymorphisms (SNPs) in the AQP4 gene that were significantly associated with functional outcomes six months post-TBI. The SNPs rs3763043 and rs3875089 were not associated with the initial severity of the injury but were correlated with patient recovery, emphasizing AQP4’s role in influencing edema and neural repair processes [236].

Elevated levels of AQP4-expressing microparticles in the bloodstream further highlighted the significance of this aquaporin in TBI. A study demonstrated that patients with severe TBI had significantly higher concentrations of AQP4-positive microparticles in arterial and cerebrovenous blood compared to healthy controls. These findings suggest that AQP4-derived microparticles could serve as biomarkers for the extent of brain injury and systemic involvement [237].

Significantly elevated AQP4 levels were reported in the CSF of patients with severe TBI compared to healthy individuals [238]. Notably, AQP4 levels tended to increase further in patients whose intracranial pressure (ICP) was successfully managed, indicating its role in the metabolic response to brain water regulation.

In AIS, AQP4 is implicated in early neurological deterioration (END) due to its association with oxidative stress and blood–brain barrier (BBB) disruption. Elevated systolic blood pressure (SBP) within 24-h of thrombolysis was directly associated with increased AQP4 levels, along with markers of oxidative stress such as malondialdehyde (MDA) and matrix metalloproteinase-9 (MMP-9). These findings suggest that AQP4 contributes to BBB dysfunction and water accumulation, exacerbating ischemic damage and clinical deterioration [239].

The pathology of sepsis-associated encephalopathy (SAE) is linked to astrocyte inflammation, which is associated with AQP4. In patients with SAE, AQP4 protein levels were found to be elevated in peripheral blood. The authors, using a cecal ligation and perforation (CLP) mouse model, suggested that learning and memory impairments in SAE were mitigated by AQP4 knockout. This effect was achieved by activating astrocytic autophagy, reducing neuroinflammation, and ultimately providing neuroprotection [172].

Finally, in patients with acute and chronic subdural hematomas, plasma levels of AQPs, including AQP2 and AQP4, have shown potential as biomarkers of disease severity. AQP2 plasma concentrations strongly correlated with chronic hematoma volume and midline shift, highlighting its significance in chronic disease processes. However, no significant correlations were observed for AQP1, AQP4, and AQP9 in acute subdural hematomas, suggesting distinct roles of aquaporins in different phases and types of brain injury [240].

Human studies in populations not treated in an ICU setting provide further evidence on the importance of AQP function in ABI. Elevated AQP4 levels in both serum and brain tissue have been demonstrated, with higher levels observed in cases of more severe injury [241]. AQP4 levels can remain elevated post-injury, indicating a prolonged response to trauma [242,243]. In addition to AQP4, AQP1 may also contribute to edema formation following TBI [244]. Postmortem studies reinforce the role of AQP4 in AIS-related edema, revealing its upregulation in astrocytes at the ischemic boundary [245,246].

#### 2.4.4. Proposed Mechanisms of Aquaporin Involvement in ABI

Human and animal studies have identified an increasing number of pathophysiological mechanisms for aquaporin involvement in ABI. These are depicted in Figure 6.

(i) Cerebral Edema Formation

AQP4 facilitates water influx into the brain parenchyma, contributing to edema after ischemia, hemorrhage, or trauma. Its upregulation in reactive astrocytes around lesion sites intensifies water transport and swelling [247,248]. In white matter, AQP4 expression increases significantly, resulting in greater swelling compared to cortical areas [246]. AQP1 has been shown to increase in astrocytes under pathological conditions, such as multiple sclerosis and cerebral infarction. This suggests that AQP1, alongside AQP4, may contribute to the astrocytic response to brain injury, possibly influencing glial scar formation and edema regulation. Notably, AQP1’s response to inflammatory cytokines differs from that of AQP4, suggesting distinct roles in CNS injury repair [249]. Comparisons of AQP4 and AQP1 expression in ischemic areas reveal that AQP4 is more prevalent, particularly in the cortex and scarred regions, and has a stronger association with basement membranes, highlighting its dominant role in edema-related water transport [250].

In SAH, AQP4 expression in astrocytes increases, especially around blood vessels, and appears to aid in water distribution across the brain’s perivascular spaces. Following SAH, blood enters the brain’s paravascular spaces, leading to vasospasm, neuroinflammation, and microvascular dysfunction. AQP4 is central in modulating water movement in these spaces, and its absence exacerbates the neurological deficits and neuroinflammation caused by SAH, as demonstrated by studies using AQP4 knockout models. This suggests that while AQP4 facilitates fluid movement, it may also play a protective role in managing inflammation post-SAH [251].

AQP1 also plays a role in SAH, particularly in edema formation around the hemorrhage site. Both AQP1 and AQP4 expression is markedly upregulated in human brain tissue following SAH, particularly on astrocytic processes, suggesting that AQP1 and AQP4 contribute to the brain’s reactive response in regulating edema [252]. Interestingly, in SAH cases, the usual polarization of AQP4 on astrocytic end-feet is lost, implying that AQP4 may be redistributed to better manage edema during acute brain injuries like SAH. However, the precise functions of AQP1 in water management post-SAH remain less understood and require further exploration [252].

(ii) Blood–Brain Barrier Disruption

Elevated blood pressure post-stroke enhances oxidative stress and AQP4-mediated BBB permeability, exacerbating edema and early neurological deterioration [239]. Several studies highlight the critical role of AQP4 in response to injury or toxic exposure. Wang et al. examined cases of methamphetamine intoxication and found increased AQP4 expression alongside elevated MMP-9 levels and reduced claudin-5 (CLDN5) expression. This suggests that methamphetamine use disrupts BBB integrity by modifying tight junctions and matrix composition, while increased AQP4 expression may serve as a compensatory mechanism to remove excess water from the brain’s extracellular space, potentially mitigating edema [72,146]. In ICH, miR-27a-3p loss deregulates AQP11 in endothelial cells, impairing BBB integrity and increasing edema [253].

(iii) Inflammatory Signaling Pathways

Aquaporins, particularly AQP4, also play a significant role in the pathophysiology of SAE, characterized by vasogenic cerebral edema and cognitive impairment. During SAE, AQP4 is upregulated in response to cerebral inflammation driven by neutrophil infiltration, which exacerbates edema [254,255,256]. AQP4 also interacts with toll-like receptor 4 (TLR4) pathways, promoting microglial activation and cytokine release in TBI and ICH [227,257]. Moreover, AQP2 expression in astrocytes drives microglial activation toward a pro-inflammatory phenotype, aggravating neuroinflammation [227].

(iv) Metabolic and Ionic Homeostasis

AQP9 contributes to lactate transport, supporting metabolic adaptation under ischemic conditions [258]. AQP4 collaborates with potassium channels like Kir4.1, balancing ionic and water transport in injured brain regions [259].

(v) Genetic Susceptibility

SNPs in the AQP4 gene, such as rs9951307 (stroke) and rs1058427 (ICH), correlate with severe edema and poor outcomes, highlighting a genetic basis for edema vulnerability [260,261]. Other AQP4 polymorphisms have also been associated with altered ICH risk and onset age, although their significance is uncertain after correction for multiple comparisons, suggesting a complex genetic influence on AQP4 expression and cerebral edema vulnerability [262].

#### 2.4.5. Aquaporins as Therapeutic Targets in ABI

Aquaporins, particularly AQP4, present promising therapeutic targets in the treatment of ABI. AQP4 inhibitors such as AER-270 and acetazolamide have demonstrated significant potential in reducing cerebral edema and improving outcomes in animal models of ABI. AER-270, a small-molecule inhibitor of AQP4-mediated water permeability, has been shown to reduce brain swelling in models of both TBI and stroke, highlighting its therapeutic potential [263].

Acetazolamide has also been investigated for its role in reducing edema in the early stages of stroke. While it has shown promise in reducing swelling, its effect on long-term recovery remains limited, indicating the need for further optimization [264].

MicroRNA-145 (miR-145) has been explored as a potential therapeutic agent for ABI. In animal models of ischemic stroke, miR-145 overexpression has been shown to downregulate AQP4, providing neuroprotection by reducing astrocytic injury and edema formation. This approach suggests that targeting the post-transcriptional regulation of AQP4 could be a novel therapeutic strategy to mitigate brain injury [265].

In the context of ICH, AQP2 expression has been linked to increased inflammation and edema. Targeting AQP2, particularly through the modulation of inflammatory pathways like TLR4/NFκB, could reduce neuroinflammation and edema, improving recovery outcomes [227]. Additionally, miR-27a-3p mimics, which target AQP11 in endothelial cells, have shown promise in enhancing BBB integrity and reducing edema after ICH [253].

Experimental models of sepsis underscore AQP4’s involvement in cognitive decline, as AQP4 deletion mitigates memory deficits, reduces neuroinflammation, and downregulates pro-inflammatory cytokines [172]. Increased AQP4 in endotoxemia-induced encephalopathy further implicates its role in SAE-related cognitive dysfunction. Collectively, these findings position AQP4 as a critical mediator in SAE pathogenesis, presenting it as a potential therapeutic target to alleviate brain edema and cognitive impairment in sepsis [266]. Moreover, research into spinal cord injury models has demonstrated that selective inhibition of AQP4 reduces edema and improves recovery outcomes, further supporting the idea that AQP4 modulation could benefit a wide range of CNS injuries [267]. These suggests that AQP4 inhibitors may have broad therapeutic potential beyond ABI, offering a strategy for managing brain and spinal cord injuries. In SAE and other inflammatory brain injuries, corticosteroids such as dexamethasone can reduce AQP4 expression by modulating inflammatory pathways. This reduction may help alleviate cerebral edema and improve cognitive function. However, the use of corticosteroids in such conditions remains controversial due to potential side effects [268].

Beyond water transport, AQP4 interacts with the glutamate transporter-1 (GLT-1), the primary astrocytic glutamate buffer. In ischemic brain tissue, AQP4 and GLT-1 have distinct spatial distributions, with AQP4 concentrated around blood vessels and GLT-1 near neurons. This coordinated distribution suggests a role for astrocytes in both water and neurotransmitter regulation, with potential therapeutic implications for targeting AQP4 and GLT-1 to enhance water and glutamate buffering in ischemic regions [269].

A connection between lncRNA-5657 and AQP4 has been identified in SAE. Inhibiting lncRNA-5657 reduced neuronal degeneration and inflammatory markers, including AQP4, suggesting neuroprotection against septic brain injury [171].

Circulating AQP4 has also shown promise as a biomarker. Baseline serum AQP4 levels in AIS patients undergoing thrombolysis inversely correlated with neurological severity and infarct size, with higher levels associated with better recovery outcomes, suggesting its potential as a prognostic biomarker for recovery [270]. In sepsis-induced delirium (SID), increased astrocytic AQP4 expression, possibly detectable through exosomes, may serve as a biomarker for SID [271].

#### 2.4.6. Summary of Aquaporins in ABI

Aquaporins are crucial in maintaining CNS water homeostasis. Their expression and regulation have been implicated in a variety of neurological and neurosurgical diseases, such as TBI, AIS, SAH, and ICH.

The dual function of AQPs in both injury propagation and recovery highlights their potential as biomarkers for disease severity and therapeutic targets in managing brain injuries and other CNS disorders. Furthermore, the identification of genetic polymorphisms and their links to clinical outcomes underscores the importance of AQPs as potential biomarkers. Ongoing research continues to explore their therapeutic potential, offering hope for improved diagnostic precision and targeted interventions. The intricate and dynamic roles of AQPs highlight their clinical significance in neuroinflammatory and vascular processes and the need for further studies to fully elucidate their therapeutic implications.

### 2.5. The Role of Aquaporins in ICU Patients with Cardiovascular Diseases

Cardiac dysfunction is a frequent and critical complication in patients with sepsis, significantly contributing to morbidity and mortality. Sepsis-induced myocardial dysfunction occurs as a result of sepsis and is often called septic-induced cardiomyopathy (SICM). It is not due to a direct infection of the heart but to the effects of systemic inflammation and the release of cytokines during sepsis. It is characterized by reduced ejection fraction and alterations in myocardial contractility. It is typically transient and reversible but can exacerbate the systemic effects of sepsis by compromising oxygen delivery to tissues [272].

In the human cardiovascular system, AQP1 is expressed in the arteries and capillary endothelium, endothelial caveolae, vascular smooth muscle cells, small muscular pulmonary arteries, cardiomyocytes, the T-tubule system, and endocardial cells. AQP3 is lowly expressed in human cardiac myocytes, AQP4 is expressed in cardiac myocytes, subendocardial and subepicardial layers, and intercalated discs. AQP7 is expressed in cardiac myocytes [273].

AQPs, particularly AQP1, are integral to maintaining cardiac water homeostasis and ion flux, rendering them essential for vascular health. Studies in AQP1-KO mice have shown that AQP1 dysfunction is linked to heart failure [57]. Until today, no studies, to the best of our knowledge, have measured AQP expression levels in critically ill patients with SICM. However, there are some experimental studies on the role of AQPs in cardiovascular dysfunction in the setting of sepsis.

In the dysfunctional hearts of aging mice exposed to endotoxin, increased AQP1 levels were seen along with increased expression of pro-inflammatory genes, suggesting that this dysregulated expression of AQP1 among others could contribute to the heart dysfunction seen in the aged mice with septic endotoxemia [274].

An in vitro model in cardiomyocytes was established using LPS, resulting in reduced H19 and AQP1 levels, accompanied by elevated miR-874 expression. H19 acted as AQP1 ceRNA in regulating miR-874 and could restore the inflammatory responses and myocardial dysfunction induced by LPS [125]. The authors concluded that H19 expression could serve as a potential therapeutic target for LPS-induced sepsis and myocardial dysfunction.

The novel AQP9 inhibitor RG100204 showed reduced septic cardiomyopathy and multi-organ failure in a murine CLP model of polymicrobial sepsis by improving outcomes like hypothermia and renal and cardiac dysfunction [275].

In an endotoxin-induced AKI rat model, the expression of AQP1 significantly increased in the LPS-treated rat hearts [212].

#### Aquaporins and Cardiorenal Interactions

As mentioned above (Section 2.3.3), Chan et al. [209] aimed to determine whether urine AQP2 can predict AKI in patients with acute decompensated heart failure (ADHF), a form of cardiorenal syndrome. They conducted a prospective, observational study in a coronary care unit, including 189 patients. Urine AQP2 demonstrated acceptable discriminative power for early detection of AKI in patients with ADHF.

Table 2 lists all the studies on aquaporins in critically ill patients with injuries involving the immune system, lung, kidney, and brain.

### 2.6. Molecular Mechanisms of AQP Regulation in Critical Illness

As already discussed, AQPs are regulated at multiple levels. AQPs are regulated by the cytokine storm, and particularly, the elevated levels of pro-inflammatory cytokines (TNF-α, IL-1β, IL-6) seen in critically ill patients [87,88,139]. Toll-like receptor (TLR) signaling during infection may also modulate AQP expression through activation of NF-κB [146,276], altering its transcription and function. The hypoxic conditions, common in critically ill patients, activate the hypoxia-inducible factor-1α (HIF-1α), which can either upregulate or downregulate specific AQPs, affecting organ-specific water balance [277,278]. Furthermore, the ROS generated during critical illness can damage AQPs directly or modulate their function through signaling pathways such as MAPK and phosphatidylinositol 3-kinase (PI3K)/Akt [146,279,280].

At the post-translational level, altered AQP phosphorylation may affect localization and function, while the proteasomal degradation of AQPs, driven by inflammatory signals, reduces their availability [146,281].

Endothelial and epithelial dysfunction can lead to impaired fluid clearance. In ARDS, AQP1 in pulmonary endothelial cells is critical for microvascular permeability and edema. Sepsis-induced endothelial damage and oxidative stress can reduce AQP1 levels, leading to impaired fluid clearance [29,173,175,176,177,178,179,180,181,182]. In the brain, AQP4 dysregulation may contribute to cerebral edema [246,247,248]. Finally, dysregulated vasopressin and angiotensin II levels in sepsis influence AQP2 in the kidney, impacting urine concentration and contributing to AKI [282].

## 3. Discussion

### 3.1. Addressing Future Challenges

#### 3.1.1. Available Products Targeting AQPs and Results in Preclinical Studies

Currently, there are no AQP-specific drugs broadly marketed. Numerous AQP modulators have been reported and patented for diagnostic and therapeutic purposes; however, their application in clinical trials has been hindered by their lack of selectivity and associated toxic side effects [220,283].

Pre-clinical inflammatory models, including CLP and LPS-induced endotoxemia, have provided valuable insights into the role of AQP regulation across various organ systems. The novel AQP9 inhibitor RG100204 is a small-molecule inhibitor shown to reduce septic cardiomyopathy and multi-organ failure in a murine CLP model of polymicrobial sepsis. More specifically, it improved outcomes like hypothermia, renal and cardiac dysfunction by inhibiting the NOD-like receptor protein 3 (NLRP3) inflammasome pathway activation and reducing inflammatory cell infiltration in lung tissue. In vitro, RG100204 effectively blocked the LPS-induced increase in hydrogen peroxide permeability and oxidative stress markers in Fao hepatoma cells [275]. Similarly, treatment of the same rodent hepatoma cell line with HTS13268, a blocker of the passage of glycerol and urea through AQP9, prevented the LPS-induced secretion of inflammatory cytokines [148,284].

The commercially available compounds DFP00173 and Z433927330 were identified as new potent and selective AQP3 and AQP7 inhibitors, respectively, [285] and were suggested to be useful in the investigation of AQPs in cytokine signaling [108]. In vitro studies in CHO cells, found no cytotoxic effects up to concentrations of 25 μM, supporting their safety profile in preclinical experiments. However, clinical translation or direct therapeutic applications have yet to be reported.

The generation of antibodies targeting the AQP channels has opened new avenues for the development of specific AQP-based therapeutics [286,287]. In an autoimmune inflammatory disease, and specifically neuromyelitis optica (NMO), an anti-AQP4 antibody blocked cell surface AQP4 binding of polyclonal NMO-IgG in patient sera in cell culture, ex vivo spinal cord and in vivo mouse models of NMO, preventing downstream cytotoxicity and NMO lesions [286,288]. An anti-AQP3 monoclonal antibody was able to inhibit AQP3-facilitated peroxide and glycerol transport in liver macrophages and prevented liver injury in experimental animal models [287]. It seems that AQP-based development of novel therapeutics to reduce inflammation in various disorders remains a promising strategy.

FDA-approved therapies include antibodies such as eculizumab, a complement inhibitor, inebilizumab-cdon, a monoclonal antibody targeting CD19, and satralizumab-mwge, a subcutaneous therapy targeting the IL-6 receptor, all of which are indicated for the treatment of neuromyelitis optica spectrum disorder (NMOSD) in adult patients who are anti-aquaporin-4 (AQP4) antibody positive [289]. Beyond NMOSD, AQPs are being investigated as therapeutic targets in conditions like cancer, edema, and metabolic disorders [290].

Table 3 summarizes the findings of preclinical studies performed with commercially available products targeting specifically AQP.

#### 3.1.2. Potential Challenges in Targeting AQPs Therapeutically

There are many potential strategies for targeting AQPs. AQP inhibitors or agonists could be potentially used to reduce edema in the lungs or brain or improve fluid clearance and maintain homeostasis in sepsis and AKI, whereas the use of AQP antagonists could prevent excessive water transport. Anti-inflammatory approaches using cytokine inhibitors, NF-κB inhibitors, or corticosteroids could prevent inflammation-driven AQP dysregulation. Anti-oxidant strategies to mitigate oxidative stress and preserve AQP function could also have a role. Finally, modifying AQP expression through gene editing or RNA-based approaches aiming at restoring their normal function in sepsis-affected organs could prove a valuable therapeutic strategy.

There are many challenges to be overcome, however. The complex regulation of AQPs depending on the organ and stage of critical illness complicates therapeutic interventions. AQPs can have protective or detrimental roles depending on the stage and type of critical illness. For instance, early inhibition of AQP4 can reduce edema, but prolonged inhibition may impair recovery. Therapeutic timing is critical but difficult to achieve in rapidly evolving conditions as in critical illnesses.

Each AQP has diverse roles across different tissues and organs. For example, AQP1 facilitates microvascular fluid transport but is also expressed in the kidneys and lungs, and AQP4 is critical for brain water regulation, but its inhibition may delay edema clearance, necessitating highly selective targeting.

The systemic modulation of AQPs may lead to undesired effects, such as altered water reabsorption in the kidney or impaired cerebrospinal fluid dynamics. Unfortunately, there are few selective AQP modulators available, and many have poor specificity, affecting multiple isoforms simultaneously. Ensuring the delivery of AQP-targeted therapies to affected organs, particularly across a disrupted blood–brain barrier or to ischemic tissues, remains a significant obstacle. The body itself may activate compensatory pathways to counteract AQP modulation, potentially reducing therapeutic efficacy.

The underlying causes of critical illness (e.g., sepsis, ARDS, AKI, ABI) involve distinct mechanisms of AQP dysregulation, necessitating tailored therapeutic approaches. Finally, the consequences of sustained AQP modulation on organ recovery and overall fluid homeostasis remain poorly understood.

Aquaporins are central to fluid regulation and organ function during critical illness. Their dysregulation contributes to pathophysiological processes such as edema, vascular leakage, and organ failure. While AQPs are attractive therapeutic targets, the complex regulation of AQPs, coupled with their ubiquitous expression and isoform-specific roles, poses significant challenges to their therapeutic targeting. A deeper understanding of AQP biology in critical illness and advances in targeted delivery and isoform-specific modulation are essential for developing effective therapies.

## 4. Conclusions

Aquaporins (AQPs) play critical roles in maintaining water homeostasis, regulating cellular function, and supporting tissue integrity, particularly in the context of critical illnesses. In conditions such as sepsis, ARDS, AKI, and ABI, AQPs contribute to fluid balance, inflammation, and cellular stress responses. The regulation of AQPs, either through gene expression changes or post-translational modifications, offers potential therapeutic targets for mitigating the effects of fluid imbalance, brain edema, and inflammation in critical care settings. Understanding the precise mechanisms of AQP involvement in these diseases and their potential role as biomarkers could lead to novel strategies for improving patient outcomes in severe, acute conditions.

## Figures and Tables

**Table 2:** Studies on aquaporins in critically ill patients with injuries involving the immune system, lung, kidney, and brain.

AQP	Disease/ Condition	Study Objective	Findings	References
AQP1	Sepsis	To study the involvement of AQP1 in immune response regulation in critically ill patients during infection acquired in the ICU. AQP1 mRNA expression was measured in leukocytes of 16 critically ill patients who develeoped sepsis and septic shock vs 13 non sepsis critically ill patients	Leukocyte AQP1 mRNA expression was induced at the onset of sepsis (median 1.71-fold increase from baseline, *p* = 0.012) and was further increased upon septic shock (median 3.00-fold increase, *p* = 0.023 from sepsis	[107]
	Sepsis	To investigate the potential mechanism of AQP1, miRNA-874, and lncRNA H19 in sepsis and the anti-inflammatory responses related to sepsis myocardial dysfunction. AQP1 mRNA expression was measured in venous blood samples of 69 sepsis patients vs 57 healthy controls	H19 and AQP1 decreased and accompanied with elevated miR-874 expression in the sepsis samples. There was a negative relationship between expression of H19 and miR-874, and a positive correlation between H19 and AQP1 expression	[125]
	ARDS	To measure macrophage MIF and AQP1 expression levels in post-mortem lung tissues samples from 15 non-smoking ARDS patients vs postmortem lung tissues from 15 age- and sex-comparable non-smoking patients who had died of non-pulmonary diseases in the ICU	AQP1 was found constitutively expressed in the alveolar endothelium, while its expression was enhanced in alveolar capillary endothelium in lung tissues from ARDS patients	[166]
	ARDS	To identify genes and/or cellular pathways involved in the pathogenesis of ICU-acquired sepsis, a genomic expression analysis of 5 polytrauma, initially non-septic patients who were admitted to the ICU was performed	Following a validation analysis, among the genes found to be dyregulated in ARDS, total blood RNA expression of AQP1 was elevated	[107]
	COVID-19 RF	To determine the relationship between inflammation and oxidative stress in COVID-19 patients. AQP1 sera levels were measured in 45 mechanically ventilated critically ill COVID-19 patients and 45 healthy individuals with negative PCR tests for SARS-CoV-2 and no chronic disease	Serum AQP1 levels were higher in the COVID-19 patient group compared to the control group (*p* < 0.01)	[167]
AQP2	AKI	To determine whether urine AQP2 can predict AKI in patients with acute heart failure	Urine AQP2 levels were higher in the AKI group; independently associated with AKI; AUC = 0.795	[209]
	TBI/Acute and chronic SDH	To investigate the potential of brain AQPs as biomarkers in 41 TBI patients	Strong correlation between AQP2 levels and the volume of chronic SDH and midline shift. In the chronic SDH group, AQP2 plasma concentration negatively correlated with the midline shift measured before surgery (rs = −0.54, *p* = 0.017) and positively with hematoma volume change between baseline and 30 h post-surgery (rs = 0.627, *p* = 0.007)	[240]
AQP3	Sepsis	To investigate whether AQPs are differentially expressed in the blood of septic patients, are related to immune cell count, and impact sepsis survival. Measured AQP3 mRNA expression in whole blood samples of 87 sepsis patients on day 1 and day 8 after sepsis diagnosis	Whole blood AQP3 mRNA expression increased over the duration of sepsis (*p* < 0.0001), AQP3 expression negatively correlated with neutrophil and leucocyte cell counts, positively correlated with lymphocyte cell count, and negatively correlated with IL-8. ROC showed that patients with AQP3 expression above the cut-off value had a higher chance of survival than those with a lower AQP3 expression on day 8 (82.4% vs. 43.8%, *p* = 0.017)	[119]
	Sepsis	To investigate the SNP rs17553719 and the expression of AQP3 in 265 sepsis patients and correlate these measurements with the outcome of sepsis patients and the release of several cytokines	CC genotype exhibited a significant decrease in 30-day survival (38.9%) compared to the CT (66.15%) and TT genotypes (76.3%) (*p* = 0.003). AQP3 mRNA expression was significantly higher and nearly doubled in the CC compared to the CT (*p* = 0.0044) and TT genotypes (*p* = 0.018) on the day of study inclusion. Increased IL-33 concentration in the CC genotype (day 0: *p* = 0.0026 and day 3: *p* = 0.008)	[120]
AQP4	TBI	To determine the time course of AQP4 variation in CSF from 20 patients receiving intensive care after TBI, and to assess the influence of increased ICP on CSF AQP4 in these patients	AQP4 significantly increased in patients with severe brain injury compared to healthy subjects (*p*< 0.002). AQP4 in CSF remained unchanged in patients with elevated ICP	[238]
	TBI	To investigate possible associations between genetic variations of the AQP4 gene and the patients’ initial TBI severity, the presence of intracranial hemorrhage, and the long-term clinical outcome after TBI in 363 patients	Significant associations with TBI outcome were detected for rs3763043 [OR (95% CI: 5.15 (1.60–16.5); *p* = 0.006], rs3875089 [OR (95% CI): 0.18 (0.07–0.50), *p* = 0.0009], and a common haplotype of AQP4 tag SNPs [OR (95% CI): 2.94, (1.34–6.36); *p* = 0.0065]	[236]
	TBI	To investigate the presence of circulating MPs of brain tissue origin in the systemic and cerebrovenous blood of 15 patients with severe TBI	Concentrations of MPs expressing AQP4 were significantly higher in the TBI group compared with healthy controls (*p* < 0.001)	[237]
	AIS	To study the association between blood pressure and the development of early neurological deterioration in 357 acute ischemic stroke patients with intravenous rt-PA thrombolysis and its possible mechanism	High SBP after thrombolysis correlates with oxidative stress-induced BBB disruption and AQP4 upregulation, linked to early neurological decline	[239]
	SAE	To investigate the effects of AQP4 associated with SAE and reveal its underlying mechanism causing cognitive impairment	AQP4 in peripheral blood of patients with SAE is up-regulated	[172]
	Acute and chronic SDH	To investigate the potential of brain AQPs as biomarkers in 41 TBI patients	No significant findings for AQP4 in acute SDH	[240]
AQP5	Sepsis	To test the hypothesis that the AQP5 promoter -1364A/C polymorphism is associated with increased 30-day survival in severe sepsis. Whole blood genotyping of 154 sepsis patients for the AQP5-1364A/C SNP	30-day survival was significantly associated with AQP5-1364A/C genotypes (*p* = 0.001). Survival rates were 57% for AA genotypes (n = 90) but 83% for combined AC/CC genotypes (56 vs. 8, respectively). AQP5-1364A/C SNP was a strong and independent prognostic factor for 30-day survival. Homozygous AA subjects were at high risk for death within 30 days [HR (95% CI): 3.59 (1.47–8.80); *p* = 0.005] compared to AC/CC genotypes	[130]
	Sepsis	To test if DNA methylation of the AQP5 promoter influences AQP5 expression, is associated with 30-day survival of septic patients, and alters NF-κB binding in whole blood samples of 88 surviving and 47 non-surviving sepsis patients	A greater methylation rate was found at cytosine site nt-937 in the AQP5 promoter linked to NF-kappaB binding in non-survivors compared to survivors (*p* = 0.002). This was associated with greater AQP5 mRNA expression in non-survivors (*p* = 0.037). Greater promoter methylation at nt-937 was also associated with an independently increased risk of death within 30 days [HR (95% CI): 3.31 (1.54–6.23); *p* = 0.002]	[101]
	Sepsis	To test if AQP5 expression and the AQP5-1364A/C polymorphism alters neutrophil migration in samples from 4 healthy volunteers	Healthy volunteer neutrophils carrying the A allele of the AQP5-1364A/C SNP show increased migration. Target-oriented migration of neutrophils was seen after 0.5 h in AA-genotype cells but only after 1.5 h in AC/CC-genotype cells, with a threefold lower migrating cell count	[112]
	Sepsis	To test if AQP5 promoter methylation differs between genotypes in specific types of immune cells. AQP5 promoter methylation was quantified in cells of 25 septic patients	The C-allele of AQP5-1364 A/C promoter polymorphism was associated with a fivefold increased promoter methylation in neutrophils (*p* = 0.0055) and a fourfold increase in monocytes (*p* = 0.0005) and lymphocytes (*p* = 0.0184) in septic patients and healthy controls. Decreased AQP5 promoter methylation was accompanied by increased AQP5 expression in HL-60 (*p* = 0.0102) and REH cells (*p* = 0.0102). The C-allele, which is associated with lower gene expression in sepsis, is accompanied by a higher methylation level of the AQP5 promoter	[132]
	ARDS	To examine the outcome associations of the AQP5 promoter -1364A/C polymorphism in whole blood from 136 patients suffering from ARDS	Patients with AA genotype of the AQP5-1364A/C SNP showed increased mortality	[168]
	ARDS	To investigate whether the AQP5-1364A/C SNP in whole blood was associated with pulmonary inflammation and survival in 136 ARDS patients	The presence of the AA genotype of the AQP5-1364A/C SNP was associated with aggravated pulmonary inflammation, while the carriers of the C allele showed attenuated pulmonary inflammation and higher 30-day survival	[27]
	AKI	To determine whether the AQP5 promoter -1364A/C polymorphism is associated with AKI in patients suffering from pneumonia evoked ARDS	On day 30, homozygous AA genotypes showed an increased prevalence of AKI compared to AC/CC genotypes (57 vs. 24%; *p* = 0.001). The AA genotype proved to be a strong, independent risk factor for predicting AKI persistence [OR (95% CI): 3.35 (1.2–9.0); *p* = 0.017]	[168]
AQP9	Sepsis	To examine the expression of AQP9 in PMNs from 14 SIRS patients and 12 healthy controls and to study the role of AQP9 in morphologic and functional changes of PMNs in SIRS	The PMNs with and without FMLP stimulation from SIRS patients showed significantly higher mean fluorescence intensity of AQP9 than PMNs from healthy subjects. AQP9 expression was significantly higher in PMNs from SIRS patients with FMLP stimulation	[115]
	Sepsis	To investigate whether AQPs are differentially expressed in the blood of septic patients, are related to immune cell count, and impact sepsis survival. Measured AQP3 mRNA expression in whole blood samples of 87 sepsis patients on day 1 and day 8 after sepsis diagnosis	AQP9 mRNA expression was not altered over the duration of sepsis. AQP9 expression positively correlated with neutrophil cell count (*p* = 0.0017) and negatively with lymphocytes and classical monocytes (*p* < 0.0257). AQP9 expression weakly correlated negatively with IL-1β, interferon-α2, and IL-33. Kaplan–Meier curve showed increased survival in patients with lower AQP9 expression (68.2% vs. 20.0%, *p* = 0.003). Elevated levels of AQP9 expression were detrimental to patient survival [HR (95% CI): 5.59 (1.58–19.56); *p* = 0.008]	[119]
	ARDS	To explore key genes and signaling pathways involved in the pathogenesis of ARDS using the mRNA expression profile dataset GSE32707, which includes mRNA expression data of 33 ARDS and 34 control samples	The initial results showed increased whole blood mRNA expression in ARDS patients. However, subsequent validation analysis in a different group of ARDS patients did not confirm these findings	[169]
	TBI/Acute and chronic SDH	To investigate the potential of brain AQPs as biomarkers in 41 TBI patients	No significant findings for AQP9 in acute SDH	[240]

Abbreviations: AIS, acute ischemic stroke; AKI, acute kidney injury; AQP, aquaporin; ARDS, acute respiratory distress syndrome; AUC, area under the curve; BBB, blood–brain barrier; CSF, cerebrospinal fluid; CI, confidence interval, FMLP, N-formylmethionyl-leucyl-phenylalanine; HR, hazards ratio; ICP, intracranial pressure; IL, interleukin; MIF, migration inhibitory factor; MPs, microparticles; NF-κB, nuclear factor kappa B; OR, odds ratio; PMNs, polymorphonuclear neutrophils; RF, respiratory failure; ROC, receiver operating characteristic; rt-PA, recombinant tissue-type plasminogen activator; SARS-CoV-2, severe acute respiratory syndrome coronavirus 2; SBP, systolic blood pressure; SDH, subdural hematoma; SIRS, systemic inflammatory response syndrome; SNPs, single nucleotide polymorphisms; TBI, traumatic brain injury.

**Table 3:** List of the most recent preclinical studies with commercially available AQP products.

Compound	Function	Findings	References
RG100204	AQP9 inhibitor	In a CLP murine sepsis model, it improved outcomes like hypothermia, renal, and cardiac dysfunction by inhibiting the NLRP3 inflammasome pathway In Fao hepatoma cells it reduced the LPS-induced increase in hydrogen peroxide permeability and oxidative stress markers	[275]
HTS13286	Blocks the passage of glycerol and urea through AQP9	In Fao cells it impaired the secretion of inflammatory cytokines	[148,284]
DFP00173	Selective AQP3 inhibitor	Anti-AQP3 mAb and DFP00173 reduced cell growth, mitochondrial respiration rate, and electron transport chain complex I activity in multiple myeloma	[285,290]
Z433927330	Selective AQP7 inhibitor	In breast cancer mice, no difference in tumor growth in either biweekly- or weekly-treated mice, decreased lung metastasis after weekly treatment with but not in the biweekly treatment cohort, and no difference in overall survival in either weekly or biweekly-treated mice	[285,291]
Anti-AQP3	Inhibits AQP3-facilitated peroxide and glycerol transport	Inhibited AQP3-facilitated peroxide and glycerol transport in liver macrophages and prevented liver injury in experimental animal models	[287]
Anti-AQP4	Targets AQP4	In an autoimmune inflammatory disesase, neuromyelitis optica (NMO), it blocked cell surface AQP4 binding of polyclonal NMO-IgG in patient sera in cell culture, ex vivo spinal cord and in vivo mouse models of NMO, preventing downstream cytotoxicity and NMO lesions	[286]

Abbreviations: AQP, aquaporin; CLP, cecal ligation and puncture; IgG, immunoglobulin; NLRP3, NOD-like receptor protein 3; NMO, neuromyelitis optica.
